# A hierarchical anti-Hebbian network model for the formation of spatial cells in three-dimensional space

**DOI:** 10.1038/s41467-018-06441-5

**Published:** 2018-10-02

**Authors:** Karthik Soman, Srinivasa Chakravarthy, Michael M. Yartsev

**Affiliations:** 1 Bhupat and Jyoti Mehta School of Biosciences, Department of Biotechnology, Indian Institute of Technology Madras, Chennai, Tamilnadu 600036 India; 2 Department of Bioengineering and the Helen Wills Neuroscience Institute, University of California–Berkeley, Berkeley, CA 94708 USA

## Abstract

Three-dimensional (3D) spatial cells in the mammalian hippocampal formation are believed to support the existence of 3D cognitive maps. Modeling studies are crucial to comprehend the neural principles governing the formation of these maps, yet to date very few have addressed this topic in 3D space. Here we present a hierarchical network model for the formation of 3D spatial cells using anti-Hebbian network. Built on empirical data, the model accounts for the natural emergence of 3D place, border, and grid cells, as well as a new type of previously undescribed spatial cell type which we call plane cells. It further explains the plausible reason behind the place and grid-cell anisotropic coding that has been observed in rodents and the potential discrepancy with the predicted periodic coding during 3D volumetric navigation. Lastly, it provides evidence for the importance of unsupervised learning rules in guiding the formation of higher-dimensional cognitive maps.

## Abstract (web summary)

Neurons in the hippocampal formation encode diverse spatial properties. Here, the authors present a hierarchical network model for 3D spatial navigation that accounts for the observed neuronal representations and predict as yet unreported cell types with planar selectivity.

## Introduction

Empirical studies in rodents show that hippocampal and parahippocampal regions contain a multitude of spatial cells that contribute to the creation of a cognitive map for navigation. Rodent hippocampus is reported to have place cells that fire at localized regions of space^1,2^. Medial entorhinal cortex (MEC) of rodents is reported to contain grid cells that activate when the animal passes through one of multiple locations arranged on the vertices of a hexagonal grid-like pattern^2,3^. Direction-sensitive cells that encode the animal’s head direction (HD) in the yaw plane are reported from a wide range of regions including post-subiculum and MEC^4–6^. Subiculum and MEC are reported to have border cells that encode the borders of the environment^7–9^.

Efforts to determine the precise coding for 3D space in rodents are ongoing, yet appeared to yield contradicting results under different behavioral conditions where they were constrained to move within a pair of orthogonal two-dimensional (2D) planes^10–14^. In parallel, results on 3D spatial maps have been obtained from bats, a mammal that naturally navigated through 3D volumetric space in unconstrained fashion during flight^15–17^. Bat hippocampus is reported to contain place cells that are active in confined 3D volumes^18^. 3D HD cells, which form an internal compass for animal’s 3D navigation, have been reported in the dorsal pre-subiculum of the Egyptian fruit bats^19^. These HD cells code for the direction of motion in terms of the three Eulerian angles viz. azimuth, pitch, and roll^19^. Grid-cell activity has thus far only been reported from the MEC of bats during 2D navigation, yet has been shown to exhibit many of the classical grid-cell features that have previously been reported in rodents, such as hexagonal firing fields and gradient in grid scale across the dorso-ventral MEC axis^17,20^. Apart from pure grid cells, bat MEC is also reported to have other spatial cells (OSCs) viz. conjunctive grid cells, pure HD cells, and border cells^20^; yet, these have thus far only been studied in 2D environments.

These rich empirical data raise difficult questions about spatial maps in higher dimensions such as: What is the learning rule for the formation the 3D spatial cells? What form of symmetry does a grid cell take in higher dimensions? What contributes to the isotropic and anisotropic coding schemes of spatial cells and why different mammals differ from each other with respect to 3D spatial coding properties? Can there exist other kinds of spatial cells to represent the space in higher dimensions? A systematic comprehensive computational model is pertinent to answer these queries. Although a significant corpus of computational models exists in the case of the 2D navigation problem^21–36^, models of 3D navigation are comparatively fewer in number. Mathis et al.^37^ treated the probable nature of grid-like representations in higher dimension as a packing problem and concluded that the periodic grid-like pattern in 3D navigation may take face-centered cubic (FCC) lattice structure^37^. A rate adaptation network model, where the grid cell is assumed to receive place-cell inputs—empirically validated in the case of 2D navigation in rodents^9,38^, but not yet in bats nor in 3D navigation—suggests the possibility of an asymptotic state of FCC or hexagonal close packing (HCP) lattice grid structure in 3D space^39^. A four ring integrator model for 3D grid cells, proposes the grid activity as a function of the co-occurrence of neuronal activity in the four distinct ring neural integrators whose reference vectors differ by 109.5°^40^, an idea that is motivated by the 2D grid-cell oscillatory interference models^22^. The model produces 3D grid cells with FCC lattice structure in 3D space. The emergence of this lattice structure could be attributed to the explicit use of reference vectors with an angular spacing of 109.5° for the ring integrators. Since the actual periodicity of the grid-cell in the 3D space has not been empirically confirmed yet, the biological validity of the chosen phase constraint on the ring integrators in the model remains to be determined. The plausible patterns of spatially periodic 3D grid cells have been extensively reviewed in ref. ^41^. With regard to the computational modeling work on HD system, Laurens and Angelaki^42^ proposed a model that gives a comprehensive multisensory framework of self-motion estimation from the vestibular signal, retinal flow, proprioception, and other sensory inputs^42^. The model also suggests relevant possibilities regarding the dynamics of the 3D HD system and raises the possibility of the gravity influence on head tilt system^43^. It also proposes a tilted azimuth model based on the dual-axis rule^44^. Analysis of the existing modeling works on the 3D spatial representation suggests a lack of unified modeling approach to account for the wide spectrum of spatial representations. Importantly, the sparsity of modeling efforts designed to address the encoding of 3D space in which most animals live in represents a major challenge to the understanding of its underlying neural computations.

To bridge this major gap, we propose here a hierarchical network model which accounts for the formation of all spatial cells reported to date in 3D space. The model shows how 3D spatial maps could be formed using unsupervised anti-Hebbian neural network while the animal follows a naturalistic complex trajectory in 3D space. The presented generalized framework not only accounts for a gamut of empirical results in 3D navigation but also makes significant predictions on the learning rule, isotropy in spatial coding and possible nature of periodic grids fields in 3D. Lastly, it also makes direct predictions for the possible existence of other kinds of novel 3D spatial representations that have yet to be reported in animals navigating in 3D. Hence, the proposed model sheds light on the principle behind the formation of 3D spatial representations across species by adopting a hierarchical systems-level modeling approach.

## Results

### Model architecture for 3D spatial cells

**Fig. 1:**
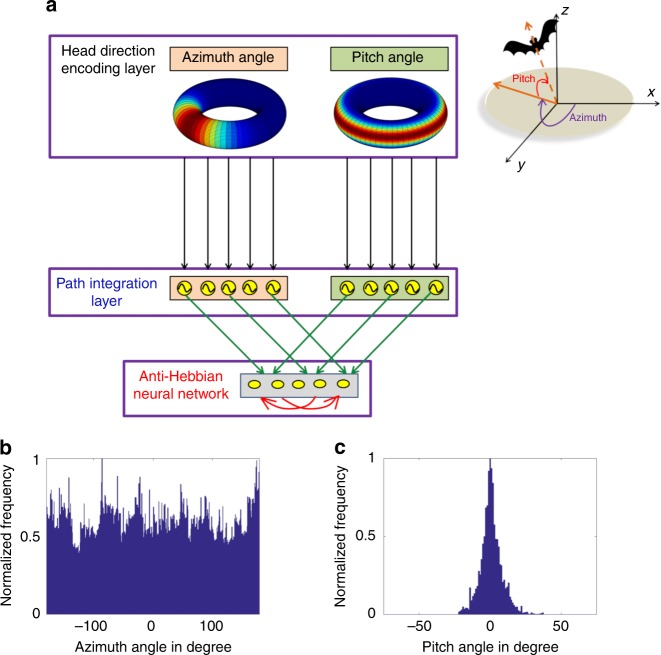
Model architecture and trajectory statistics. **a** The architecture of the proposed model. Illustration of the hierarchical nature of the model starting from the head-direction encoding layer with parallel layers that code for the azimuth and pitch angle. Inside the head-direction encoding layer, toroidal representations for pure azimuth (left) and pure pitch (right) are shown. Head-direction layer ensures one-to-one connectivity with the corresponding oscillatory path integration layer which further converges to the anti-Hebbian network. Green arrows indicate the afferent synaptic weight connections that are trainable by Hebbian rule and the red arrows indicate the lateral inhibitory synaptic connections that are trainable by Stent rule. Top right side to the figure shows the depiction of the pitch and the azimuth angle in the 3D Cartesian coordinate system. **b** Angular distribution of the azimuth direction of the simulated trajectory. Azimuth angle of the simulated trajectory is uniformly distributed. **c** Angular distribution of the pitch direction of the simulated trajectory. Pitch angle of the simulated trajectory has a narrow range as shown in the figure with a Gaussian distribution (mean (*μ*) = 0, variance (*σ*^2^) = 58.25)

The proposed model is driven by considering the movement of the virtual animal during its active exploration in the 3D space (see Supplementary Note 1). Simulated flight trajectories concur with the empirical trajectory of bats in terms of its azimuth and pitch distribution statistics^18,19^. Azimuth angle is sampled from a uniform distribution which spans the complete angular space (Fig. 1b). However, since the animal’s flight is devoid of sharp dives and ascents, its pitch exhibits a narrower range than azimuth (Fig. 1c).

The model adopts a hierarchical architecture comprising of: HD encoding layer, path integration layer, and finally lateral anti-Hebbian neural (LAHN) layer (Fig. 1a). Empirical studies in bats report the existence of separate neural ensembles to code for the three Eulerian angles—azimuth, pitch, and roll, with the roll-coding population comprising a non-significant population^19,42^. Conforming to this empirical result, HD layer in the proposed model has two parallel layers viz. azimuth and pitch direction encoding layers. Roll cells are not considered in the proposed model. This is because previous studies^42,44^ have shown that sufficient combinations of pitch and yaw angle could account for any rotation in the 3D space. Hence, 3D direction could be encoded even in the absence of explicit roll-coding cell. Since azimuth cells are higher in distribution compared to pitch cells^19^, they are taken in a ratio of 7:3 in the model, as reported empirically in the bat^19^. Irrespective of this distribution, the preferred directions of both cell types span the complete 360° angular space. Hence, in the model, these cell types compute the projection of the current heading direction and forward pass the information to the downstream path integration layer. Like HD layer, path integration layer also splits into two parallel layers of azimuth and pitch, respectively, which ensures a one-to-one connectivity with the upstream HD layer. The path integration layer is an array of low-frequency phase oscillators, compatible with reports in bats (0.5 Hz, see refs. ^18,20,45^) where path integration in achieved by integrating the afferent encoded direction and speed information of the animal’s flight into the phase of the oscillator.

In the final hierarchy of the model, the information from the parallel pathways (azimuth and pitch pathways) converges and forms the afferent input to the anti-Hebbian network. Hence LAHN receives the integrated information of the 3D space in which the animal navigates. LAHN is a recurrent neural network whose afferent weight connections are updated using Hebbian rule, that is, higher correlation of pre-synaptic and post-synaptic neural activity strengthens the synaptic weights between them^46^. Lateral recurrent connections are updated using anti-Hebbian rule, that is, higher correlation of pre-synaptic and post-synaptic neural activity suppresses the synaptic weights between them^46^. From now on, we call anti-Hebbian rule as Stent rule, after the original formalization of this rule^47^. Hence, the lateral connections induce competition among the neurons, whereas the afferent Hebbian connections enable extraction of principal components (PCs) from the input data^48,49^. Such an iterative learning network also has the advantage of analyzing the temporal evolution of the spatial cells. Owing to its generalized architecture and the local learning rules, LAHN qualifies as a biologically plausible neural network.

After training the model, the LAHN neural activity is tracked along realistic 3D flight trajectories of the animal. Whenever the activity of a neuron crosses a threshold value, we assign this threshold crossing moment an action potential, which allows us to compute the firing fields of that particular neuron.

### Emergence of 3D place fields

**Fig. 2:**
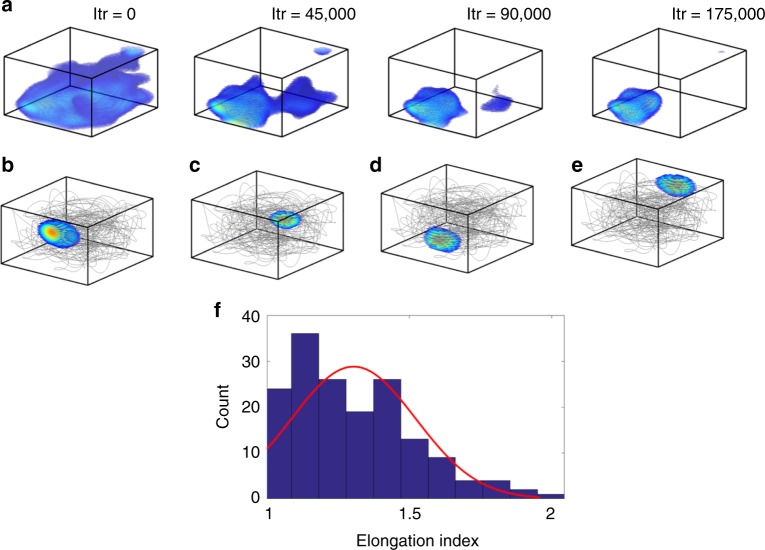
Emergence of 3D place cells and their isotropic nature. **a** Figure, from left to right, shows the snapshots of the temporal evolution of the localized volumetric 3D place-cell firing field of a LAHN neuron that resembles the empirically reported 3D place cells. Iteration number (Itr) is given above each box. **b**–**e** show the rate maps of four example 3D place cells from the trained model, overlaid on the flight trajectory traversed by the animal (gray lines). It is evident from the figure that the place cells code for different regions of the space. **f** The distribution of the elongation index of the fitted ellipsoids results in similar values to those observed empirically in freely flying bats^18^. Elongation index is the descriptor of the isotropic nature of the firing map. The Gaussian curve fitted to the distribution (red curve) mainly shows the emergence of isotropic nature of place cells from the model (mean (*μ*) = 1.3042, standard deviation (s.d.) = 0.2179). Approximately 65% of place cells from the model exhibit isotropic place fields

While training the LAHN, 32.43% neurons evolve firing fields that eventually occupy a confined volume in the 3D space (Fig. 2a). Place cells from the CA1 region of bat hippocampus are reported to have isotropic firing fields, that is, the firing fields show equal variance in all configurations of three orthogonal dimensions^18^. Compatible with these empirical results, we find that once the LAHN network converges, the emerging firing fields resemble the empirically reported volumetric place fields in flying bats (Fig. 2b–e). To explicitly compare the place fields evolved from the model with their empirical counterparts, we compared their isotropic index (*ξ*), which is derived by fitting an ellipsoid to the 3D firing field^18^. *ξ* is the ratio of the largest to the smallest axis of the fitted ellipsoid. The Gaussian distribution fitted on the *ξ* shows that a significant number of simulated neurons (65.25%) exhibit isotropic firing fields (mean = 1.3042, s.d. = 0.2179) (Fig. 2f). (Elongation index threshold is taken as 1.38 from a shuffling analysis, see Supplementary Note 2a.)

### Emergence of 3D spatial periodicity

In the bat experimental literature, HD and place cells are the only two spatial cells reported while the animal navigates in 3D space^18,19,50^, whereas grid cells have only been recorded in animals crawling on 2D planes^20^. Although empirical studies for 3D grid cells have been performed in rodents^51,52^, the existence of 3D periodic spatial representations is still unresolved. Nevertheless, predictions for the existence of such representations can emerge from the proposed model, as we shall show next.

**Fig. 3:**
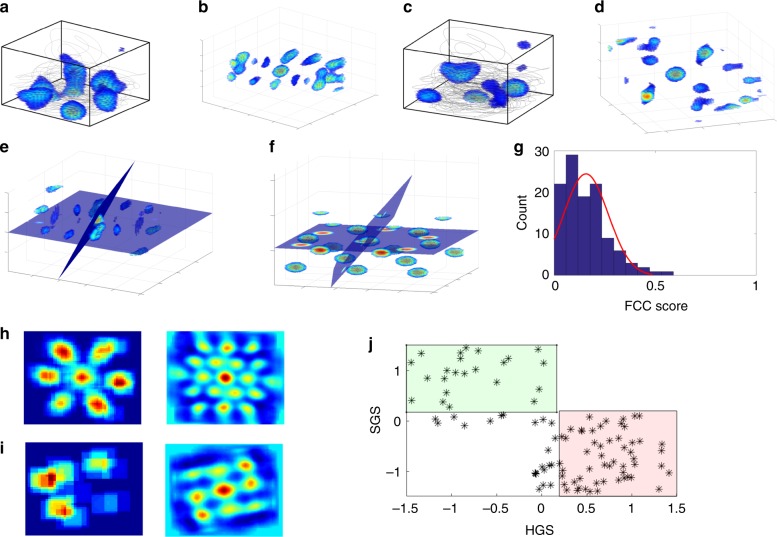
Emergence of 3D spatially periodic neurons and their symmetry. **a** 3D firing map of a LAHN neuron overlaid on the animal’s flight trajectory. **b** 3D autocorrelation map of the same neuron computed to observe the inherent periodicity of the spatial representation. **c** 3D firing map of another LAHN neuron overlaid on the animal’s flight trajectory. **d** 3D autocorrelation map of the same neuron computed to observe the inherent periodicity of the representation. Two neurons (**a** and **c**) are purposefully taken to show the difference in their periodic representations which will be made clearer through figures **h**, **i**. **e** Two planes at 72° transecting through the central peak of the volumetric autocorrelation map of a spatially periodic LAHN neuron. This is done to check for any FCC symmetry in the 3D grid representation of that neuron. **f** Two planes at 72° transecting an analytically simulated FCC lattice structure through its central peak. **g** Distribution of the FCC scores of the spatially periodic neurons from the model. The Gaussian curve fitted to the distribution (red curve) shows that the neurons have less tendency to form the FCC symmetry (mean (*μ*) = 0.1575, standard deviation (s.d.) = 0.1098). **h** (Left) Projection of the 3D firing map shown in **a** onto the XY plane which shows hexagonal rate map. (Right) Autocorrelation map of the 2D firing map to quantify the 60° periodicity. (**i**) (Left) Projection of the 3D firing map shown in **c** onto the XY plane which shows square rate map. (Right) Autocorrelation map of the 2D firing map to quantify the 90° periodicity. **j** Scatter plot of hexagonal and the square gridness scores of each neuron (marked as star) on a 2D plane. The red area represents the hexagonal grid regime (55%; hexagonal gridness threshold value is 0.1686), the green area represents the square grid regime (23%; square gridness threshold value is 0.1952), and other neurons whose gridness scores are not significantly higher than the prescribed thresholds (star marks outside the green and red box) do not belong to hexagonal nor square symmetry

Apart from the spatially localized activity of the aforementioned place cells, LAHN neuronal ensemble shows spatially periodic activity too. Spatially periodic activity is evident from the rate maps of the grid neurons (Fig. 3a, c). However, autocorrelation maps are computed to analyze the symmetry of the grid periodicity in higher dimensions (Fig. 3b, d). Previous modeling studies have predicted the possibility of FCC lattice structure for the grids in the 3D space owing to its higher packing fraction^37,39^.

FCC is a cubic lattice structure that results from stacking hexagonally arranged layers of spheres one above the other^53^. Considering three layers A, B, and C where B and C are the translations of A, the sequence ABCABC results in an FCC structure^53^. In FCC symmetry, one can find four planes, at an angular difference of 72°, transecting the center of FCC, and each plane has peaks arranged in a hexagonal fashion^39^. An ideal FCC lattice structure can be analytically simulated by a linear combination of four 3D waves whose wave vectors are at angles of 109.5°^39,40^.

In FCC symmetry analysis of 3D grid fields, initially the autocorrelation map is transected into many slices that pass through the origin. Hexagonal gridness score (HGS) of each slice is computed. The slice with the highest gridness score is taken as the reference plane. As per Stella and Treves^39^, FCC lattice structure has apparently three more planes with hexagonal symmetry at 72° from the reference plane and among one another (Fig. 3e shows two such planes; see Supplementary Note 3 for the explanation on the generation of the transection planes) and an average of top three gridness scores of those planes is computed. A similar procedure is performed on the analytical FCC too (Fig. 3f). An FCC score is computed by taking the ratio of these two average gridness scores (analytical FCC in the denominator). Negative average gridness score (if any) is set to zero so that FCC score will be between 0 and 1. Hence, if the spatial periodicity is close to FCC symmetry, the FCC score will be ~1. The aforementioned analysis on the LAHN spatially periodic neurons shows that the grid neurons from the model apparently do not show FCC symmetry (Gaussian distribution fitted on the FCC score has mean = 0.1575 and s.d. = 0.1098) (Fig. 3g).

Since the spatially periodic neurons from the model show less tendency towards the FCC structure (mean is <50% of the maximum value of FCC score), to check the symmetry adopted by the grids in the model, rate maps are projected on to the three major orthogonal planes *XY*, *YZ*, and *XZ*, respectively (Fig. 3h, i). Sixty degree rotational symmetry is checked on each plane by computing their HGS^3,21^. Along with the HGS, square gridness score (SGS) is also computed to consider the possibility of 90° planar symmetry^21^. A gridness score greater than the threshold value (obtained by shuffling analysis; see Supplementary Note 2b) on any one of the planes means that the grid cell shows a planar symmetry in the 3D space. Scattering the computed gridness scores on a 2D space of HGS and SGS (Fig. 3j) confirmed the planar symmetry of the spatially periodic neurons from the model. The critical information conveyed by this figure (Fig. 3j) is that the grid activities of the simulated neurons show both square and hexagonal symmetry with a preference for the hexagonal symmetry (55%), and, although a minority (7%), the remaining neurons evolve neither square nor hexagonal symmetry in the model.

In the experimental study, when the rat forages on a 2D plane (*XY*), place cells exhibit isotropic localized firing fields and grid cells exhibit hexagonal representations. Yet, when the rat is made to climb over a pegboard (vertical *YZ* plane), the spatial representations of the same cells are reported to transform into an anisotropic representation, manifested as a single stripe for most place cells and multiple stripes for the grid cells^52^. This intriguing transformation exhibited by spatial neurons is still an enigma. This enigma is further enhanced by the empirical data obtained in bats. Place and grid cells of bats exhibited similar spatial firing patterns to those of rodents when the bats were navigating in 2D environments (Yartsev et al.^21^). However, unlike anisotropic place fields in rodents moving along the *z*-axis, place cells of flying bats are reported to be isotropic (Yartsev and Ulanovsky^18^). Since 3D grid cells have not been empirically reported from flying bats so far, nothing conclusive can be stated with regard to its isotropic nature. The proposed model probes this problem of anisotropy and the reason behind this transformation. Specifically, we postulate that because the movement patterns of rodents and bats on 2D planes are far more similar to each other than in 3D space, these differences in spatial movement patterns might lie at the core of the differences in neural coding. Such notions have been expressed before, yet never actually tested^18^.

**Fig. 4:**
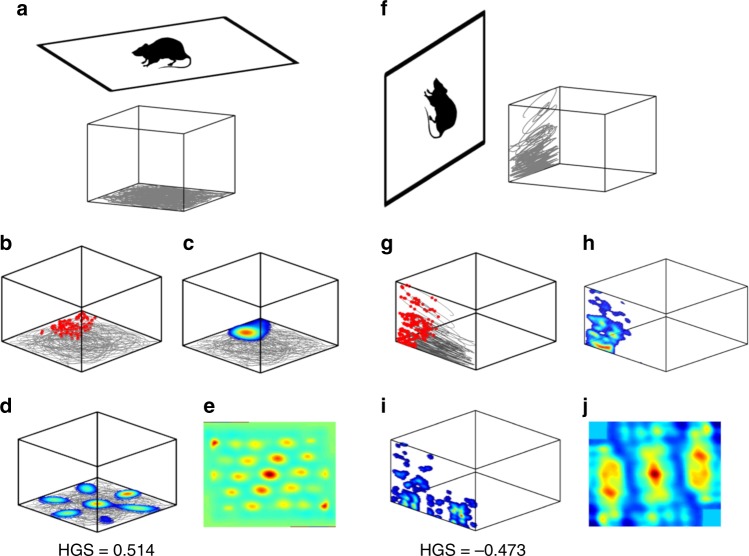
Horizontal and vertical anisotropy in grid cell coding. **a** A cartoon of a virtual animal navigating on horizontal *XY* plane (top) and the trajectory traversed by the animal (bottom). **b**, **c** Raw data (movement trajectories in gray and action potentials in red) and firing rate map of a LAHN place neuron in the model showing localized isotropic representation on the horizontal *XY* plane. **d**, **e** Firing rate and autocorrelation map of a LAHN grid neuron in the model showing hexagonal representations on the horizontal *XY* plane (HGS > HGS_thresh_). Hexagonal periodicity is evident from the autocorrelation map and the gridness score (HGS). **f** A cartoon of a virtual animal navigating on vertical *YZ* plane (left) and the trajectory traversed by the animal (right). **g**, **h** Raw data (movement trajectories in gray and action potentials in red) and firing rate map of the same LAHN place neuron in the model showing stripe-like representations on the vertical *YZ* plane. **i**, **j** Rate map data (movement trajectories in gray) and autocorrelation map of the same LAHN grid neuron in the model showing stripe-like representations on the vertical *YZ* plane (HGS < HGS_thresh_). Stripe periodicity is evident from the autocorrelation map

To achieve this, two different trajectories are simulated on two different planes, one on horizontal and the other on a vertical plane (Fig. 4a, f). Since, thus far, only place cells have been recorded in both species, we begin our investigation there. When the virtual animal navigates on the horizontal *XY* plane (Fig. 4a), the model gives rise to localized isotropic place fields (Fig. 4b, c) akin to classical place cells. When the animal is made to navigate on the vertical *YZ* plane (Fig. 4f), the previously formed isotropic firing field stretches out and forms stripe-like firing field (Fig. 4g, h). Grid neurons in the network exhibit hexagonal firing fields when the virtual animal navigates on the horizontal *XY* plane (Fig. 4d). When the plane of navigation is switched from horizontal to vertical, it results in a transformation of the firing fields from hexagons (Fig. 4d) to multiple stripes (Fig. 4i). These symmetries are also reflected in the autocorrelation maps of the respective firing fields (Fig. 4e, j).

Emergence of spatial representations in the proposed model is a result of the projection of path integration values on the weight vectors that maximize the variance of the output. It is important to emphasize here that the animal is not retrained on the vertical plane i.e. it is the same LAHN weight vectors (trained for horizontal navigation) that are used for the navigation on both planes and exhibit different coding schemes. Hence, we hypothesize that the absence of hexagonal representations on the vertical dimension may not be a network property and could be attributed to the lesser range in the vertical pitch distribution (Fig. 1c). If this is the case, change in the range of pitch distribution should reflect a corresponding change in the grid-cell anisotropy too.

**Fig. 5:**
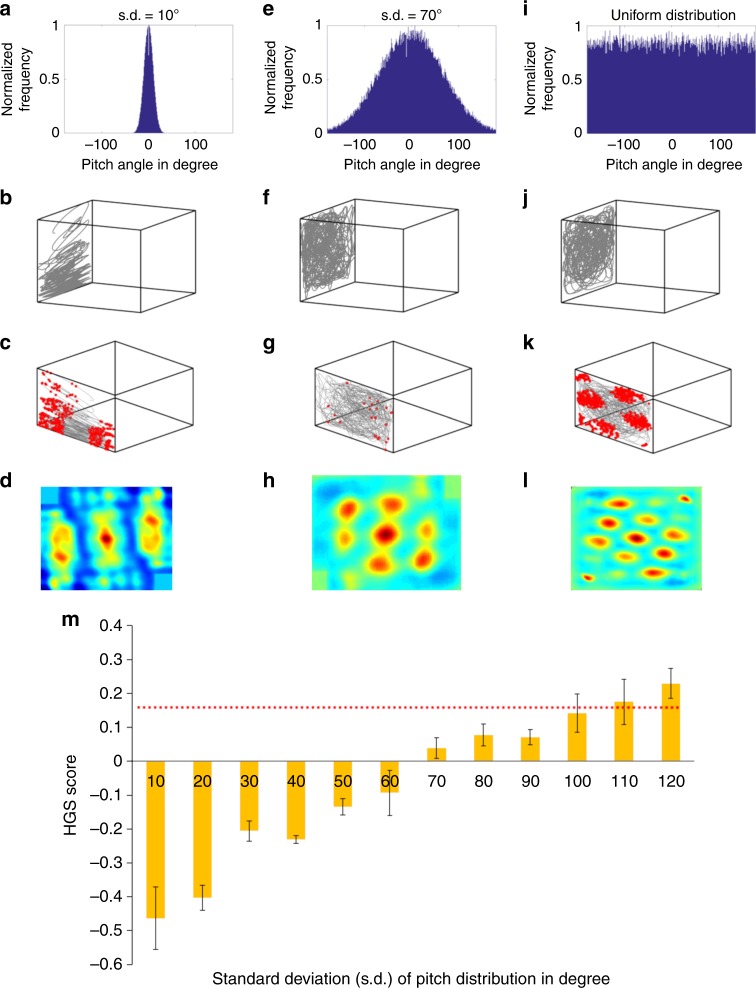
Influence of pitch range on grid cell anisotropy. **a** Skewed pitch distribution with mean (*μ*) = 0; standard deviation (s.d.) = 10°. **b** Trajectory simulated on the vertical wall using the skewed pitch distribution as shown in **a**. **c** Stripe-like firing fields of the LAHN neuron formed on the simulated vertical plane trajectory. **d** Autocorrelation map of the representation that clearly shows the stripe periodicity. **e** Less skewed pitch distribution with *μ* = 0; s.d. = 70°. **f** Trajectory simulated using less skewed pitch distribution. **g** Firing fields of the same neuron formed on the vertical plane trajectory. Loss of stripe-like representation is evident from the firing field. **h** Autocorrelation map of the representation that shows 60° grid symmetry. **i** Uniformly distributed pitch distribution. **j** Trajectory simulated using the uniform pitch distribution that gives the animal the leverage to choose any direction on the vertical plane. **k** Hexagonal firing fields of the neuron on the vertical plane trajectory formed from uniform pitch distribution. **l** Autocorrelation map of the same firing fields that shows cogent hexagonal grids. **m** Dependence of the 60° rotational symmetry of the neural representation formed on the vertical plane (quantified as HGS on the *y* axis) to the s.d. of the pitch distribution of the animal’s trajectory on the same vertical plane. A significant increase of HGS score (i.e., crossing the HGS_thresh_ shown as horizontal red dotted line) is evident at 110° s.d. (critical angle)

To test this hypothesis, three different trajectories with different pitch ranges are considered, such as: trajectory with restricted motion on the vertical plane (Fig. 5b) due to highly skewed pitch distribution (Fig. 5a), trajectory with less restricted motion on the vertical plane (Fig. 5f) due to an increase in the pitch range compared to the first one (Fig. 5e), and an unconstrained trajectory on the vertical plane (Fig. 5j) with uniform pitch distribution (Fig. 5i). Since both grid and place neurons in the LAHN undergo similar stripe-like transformation, the proposed hypothesis is analyzed only on one type of spatial cell, that is, grid cell (grid representation can also be quantified using the gridness score). LAHN grid neurons exhibit spatial periodicity on the vertical plane in all the three cases (Fig. 5c, g, k), but the neural representations are different for each case. As the pitch range increases, the spatial representations on the vertical plane gradually transform from stripes to hexagons, which is evident from the respective neural firing fields (Fig. 5c, g, k) and the autocorrelation maps (Fig. 5d, h, l).

To obtain a conclusive result on the relation between the pitch range and the grid formation on the vertical plane, pitch distributions with standard deviation (s.d.) ranging from 10° to 100° (with a step size of 10°) are considered. Average HGS of spatial representations formed on trajectories from each pitch distribution is plotted against the respective s.d. (Fig. 5m). The HGS score takes a value greater than the threshold (shown as horizontal like in Fig. 5m) for 110° s.d. (Fig. 5m). We call this the critical angle at which the stripe representations get transformed to significant hexagonal representations. This aspect is addressed again in the Discussion section.

In the previous case where the animal climbed over a vertical wall with a varying range of pitch values, the firing fields exhibited an intriguing grid transformation from stripes to a more optimal hexagonal representation (optimal on a 2D surface). Now, we ask if there is a possibility for the 3D grid representation to transform from planar to a more optimal FCC representation while the animal is less constrained in its 3D navigation? To answer this, we vary the range of the pitch values while the animal performs a complete 3D volumetric navigation.

**Fig. 6:**
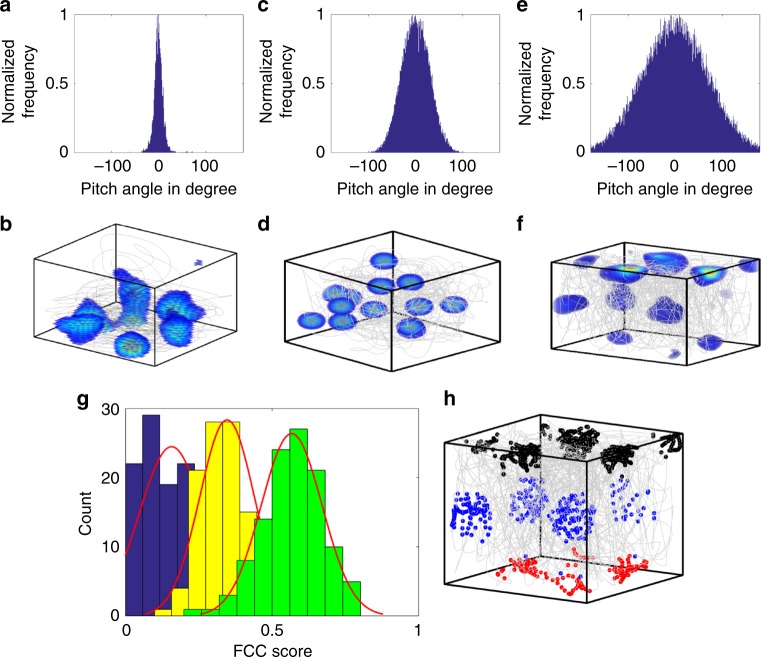
Variation of the 3D grid representations during volumetric navigation. **a**, **c**, **e** Three different pitch angle distributions with s.d. = 10°, 25°, and 75°, respectively. **b**, **d**, **f** Rate maps of the same neuron for the three different trajectory statistics. **g** The distribution of the FCC score of neural representations for the three different trajectory statistics (color coded as blue (for pitch distribution with s.d. = 10°), yellow (for pitch distribution with s.d. = 25°) and green (for pitch distribution with s.d. = 75°)). It is evident from the distribution that as the pitch angle range increases, the grid representation moves closer to the FCC regime. **h** Firing field of a neuron that shows maximum FCC score (~0.8). The firing fields are presented with three different colors to show the ABC sequence present in the grid representation which is a unique feature of FCC lattice structure

We initially computed the FCC score of the periodic neural representations while the animal flies with a skewed pitch distribution (s.d. of 10°) (Fig. 6a). Since varying the pitch distribution in a continuous fashion and computing the FCC score is computationally expensive, we consider two other flight trajectories with contrasting pitch distribution (i.e., one with 25° s.d. (Fig. 6c) and the other with 75° s.d. Figure 6e). If the grid representations show a tendency to move towards the FCC regime, it should be evident from these two contrasting pitch distributions. In doing so, we find that by increasing the range of the animal’s flight trajectory pitch distribution, the corresponding 3D rate maps of the spatially periodic neurons in the model (Fig. 6b, d, f) show a tendency to shift towards FCC representation, as evident from the histogram and the corresponding Gaussian fit (Fig. 6g). This result along with the one shown in Fig. 5 reinforces the dependence between the neural spatial representations and the fine details of the animal’s trajectory statistics. Considering the current result and also the higher packing ratio of FCC structure (compared to the other 3D lattice structures)^37,39,40^, it is quite likely that the grids exist in an FCC form in 3D space. However, we do not neglect the possibility of the existence of other kinds of grid representations as mentioned in ref. ^41^. We will return to this notion in the Discussion section.

### Emergence of 3D border and plane cells

**Fig. 7:**
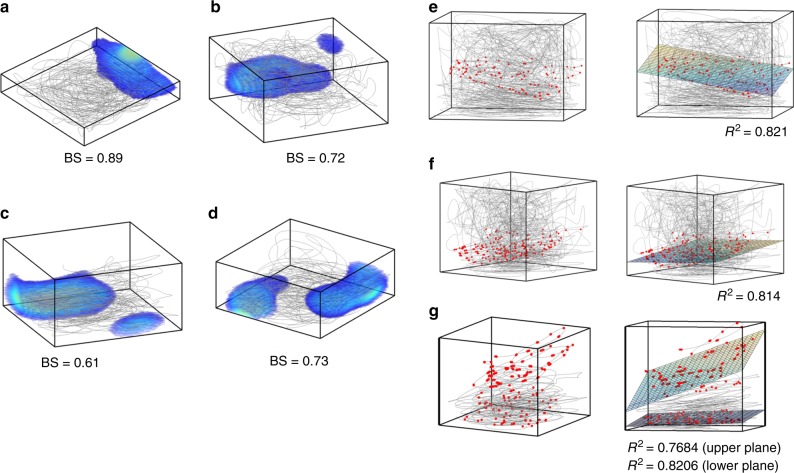
Emergence of 3D border and 2D plane cells. **a**–**d** 3D rate maps of four LAHN neurons that show affinity towards the 3D borders or walls of the box. Each 3D border cell has its own preferred border (either single or double preferred borders) to fire akin to the 2D border cells in the rodents. Border score (BS) (average of top two BS) of each neuron is shown near to each box. **e**, **f** (Left) Raw data (movement trajectories in gray and action potentials in red) of three neurons that activate predominantly on a 2D plane in 3D space, that is, they are coding for a lower-dimensional subspace and hence termed as plane cells. (Right) 3D plane fitted to each plane cell firing field to quantify their planeness in terms of the *R*^2^ value of the fitted plane. **g** (Left) Raw data (movement trajectories in gray and action potentials in red) of a neuron showing activity on multiple 2D planes and termed as stack cells. (Right) Two 3D planes fitted to the upper and lower firing fields. *R*^2^ value of each plane is shown near to the box

Border cells convey information about the borders of the environment in which the animal navigates. These types of cells are reported from the rodent MEC and pre-subiculum and post-subiculum^7–9^. However, similarly to 3D grid cells, the description of 3D border cells has yet to be provided empirically as these are thus far only reported in animals moving on 2D planes^7–9,20^. Here, we report the possibility of the existence of border-like activity even in higher dimensions. After training the model, some LAHN neurons start to exhibit higher activity near the borders of the environment (Fig. 7a–d). This border related activity is quantified by calculating the border score (BS) of the respective neuron on each orthogonal plane. For a neuron to qualify as a 3D border cell, the BS on any two major orthogonal planes should exceed a threshold value (the threshold value is chosen based on the shuffling analysis, see Supplementary Note 2c). To compute the BS, we adopted the same approach as in the case of 2D navigation, that is, the ratio of the difference between the extent of a single field on any wall and the average distance to the nearest wall of each bin in the rate map (weighted by its activity) to the sum of these quantities^7,9,54^. Hence, the model predicts the possibility of the existence of border cells in 3D navigation.

Our model also revealed the possibility of a previously undescribed spatial cell type in 3D space which codes for 2D surface while the animal performs 3D navigation. We call these neurons plane cells. Some LAHN neurons show this type of coding where neuronal firing fields are arranged on a 2D plane in a 3D space (Fig. 7e–g (left)). The amount of planeness of a cell is quantified by the *R*^2^ value of the plane fitted to the 3D firing field (Fig. 7e–g (right)). *R*^2^ value, which is the proportion of the variance in the dependent variable predictable from the independent variable, ranges from 0 to 1. The border cells also can have high plane index (PI) since it is also coding for a plane (border), or an entire face of the navigation volume. Hence, for a cell to qualify as a pure plane cell it should have high *R*^2^ value (>0.7528, see Supplementary Note 2d) and less BS value (<0.5228). Apart from the plane cells that have a single plane of firing field, we also observed LAHN neurons with multiple planes of firing fields (Fig. 7g) and termed them as stack cells. We believe that this model prediction on the possible existence of plane cells or stack cells is critical because coding of lower-dimensional subspace (a plane) while performing navigation in higher dimensions (3D space) may be functionally relevant for animals navigating in 3D volumetric environments. For instance, a horizontal plane cell can convey the information about the altitude of the animal’s flight from the ground which is critical for the efficacy of its navigation.

### Statistical analysis of cell types from the model

**Fig. 8:**
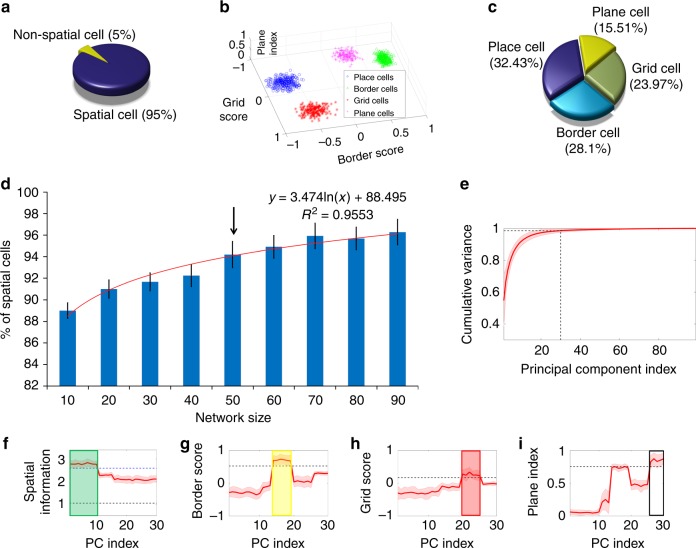
Distribution of cell types and the influence of network size on the model. **a** The Pie chart shows the distribution of spatial cells and non-spatial cells from LAHN network with 50 neurons. Cells are classified as spatial cells based on their spatial information (SI) index. Values are averaged over 20 trials (i.e., training the LAHN 20 times). Distribution shows that most of the neurons in the network after training code for one or the other spatial variable. **b** Clustering of different spatial cell types into place cells, border cells, grid cells, and plane cells in the descriptor space (grid score, border score, and plane index). **c** Pie chart shows the distribution of different spatial cell types from LAHN network with 50 neurons. Distribution shows that out of the 95% of spatial cells formed, 32.43% emerge as place cells, 23.97% emerge as grid cells, 28.1% form border cells, and 15.5% form plane cells. **d** Graph (mean ± s.d.) shows the influence of LAHN size on the distribution of spatial cells. The logarithmic trend line (red curve) shows that the percentage of average spatial cell distribution increases as the size of the network increases. The black arrow indicates the network size from which the calculations in **a**–**c** are drawn. **e** Shaded plot of the cumulative variance graph of the principal components. Intersection of the black dotted lines shows the coordinate points at which the cumulative variance reaches 99% at 30th principal component. **f**–**i** Variation of spatial cell descriptors such as spatial information index, border score, grid score, and plane index, respectively, with respect to the principal component (PC) index. (For e.g. PC index 1 represents the first principal component and so on and so forth.) The dotted line in each figure represents the threshold value of the respective spatial descriptor. In **f**, the black dotted line indicates the spatial information index threshold and the blue dotted line indicates the threshold value above which the cell qualifies as a place cell

Based on indices such as spatial information (SI), grid score (GS), BS, and PI, a quantitative analysis is done to account for the distribution of cell types that emerge from the model. Initially the distribution of spatial and non-spatial cells in the model is computed based on the SI index of each LAHN neuron. Based on the SI index, 95% of LAHN neurons evolve as spatial cells (based on average SI obtained after retraining the LAHN for 20 times; see Supplementary Note 2e) and remaining 5% as non-spatial cells (Fig. 8a). This means that the network is able to code for one or the other spatial variables like place, grid, border, plane, and so on. Further analysis is done to check the distribution of the spatial cells formed from the model. The spatial cells are clustered in the spatial descriptor space based on their threshold values (Fig. 8b). The distribution values convey that, out of the 95% spatial cells formed from the model, 32.43% are place cells, 23.97% are grid cells, 28.1% are border cells, and 15.5% are plane cells (Fig. 8c).

Network size is a critical factor in the present modeling study. Previous modeling works on grid cells in 2D environments have also shown the criticality of this factor^23^. Hence, a range of network sizes (10 to 90 neurons with a step size of 10) are considered and each network is trained 20 times to check the percentage of spatial cells formed for each time. As in the previous case, spatial cells are defined based on their SI index. Here, we do not account for the explicit spatial cell types; instead, we are interested in the influence of network size on capturing any spatial variable. The trend line shows that as the size of the network increases, it accounts for more spatial features and form more spatial cells (Fig. 7d). However, there is an upper bound on the size of the network that can be used in the current model. This is because of the learning rule of LAHN. It has been previously proven that if the afferent and the lateral connections of a network are trained using Hebbian and Stent learning rules, respectively, weights in such a network converge to the subspace spanned by the PCs of the input data^46^. Hence, LAHN can be considered as a neural network implementation of PC analysis (PCA). PCA is an orthogonal linear transformation that rotates the data to the maximal variance direction and hence performs dimensionality reduction of the input with minimal loss of information^55^. Taking this into consideration, LAHN represents the path integration inputs with minimal number of neurons where each neuron specializes to code for some unique feature of the space and eventually turns out into one of the spatial cell types as shown above. Hence, the maximum network size that can be implemented using LAHN is *N* − 1, where *N* is the total number of path integration neurons. If the network size is more than *N* − 1, the network carries redundant information which is not an optimal way of coding any stimulus.

### Formation of spatial cell bands on the eigen spectrum

The LAHN network has been previously shown as a neurally plausible implementation of PCA^46^. Here, the analysis of LAHN network shows that for a network size of 50 neurons, around 95% cells extract some spatial features and qualify themselves as spatial cells, by virtue of their high SI index (Fig. 8a). The spatial cell types which we consider here such as place cells, border cells, grid cells, and plane cells show a defined way of distribution among themselves (Fig. 8c). To analyze the reason behind this, a direct PCA is done on the input path integration matrix. The cumulative variance graph shows that the first 30 PCs (out of 100) capture ~99% of the input variance (Fig. 8e). Hence, further analysis is done only using the first 30 PCs. Spatial descriptors such as SI, BS, GS, and PI are computed for the rate maps obtained from the projections of the path integration values on to each PC. Graphical plot of each descriptor (averaged over 35 trajectories) with respect to the PC index (up to first 30 PCs) shows a clear band of spatial cell distribution in the eigen spectrum (Fig. 8f–i). Place cells that show high SI index (because of their localized activity) show a band in the high variance side of the eigen spectrum (green box in Fig. 8f). Border cells whose BS are greater than the border threshold occupy the middle band of the eigen spectrum (yellow box in Fig. 8g). Grid cells that show positive GS occupy their position next to the border-cell band in the eigen spectrum (red box in Fig. 8h). Plane cells whose PI crosses the plane threshold come next in the eigen spectrum (white box in Fig. 8i). We want to mention here that apparently there also exists a separate independent band between the place and the border regime on the eigen spectrum. This band qualifies to be a spatial cell since the SI index of this band exceeds the SI threshold (black dotted line in Fig. 8f). However, they do not pass the other spatial descriptor criteria such as GS, BS, and the PI score. Owing to its SI index, we name this as OSC band. This finding is reminiscent of the result from Diehl et al.^54^, where neurons in the MEC of the rat can exhibit a high SI index but with atypical non-grid activity structure^54^. It may be possible that OSC bands, even though not yet empirically tested, may represent such non-grid spatial cells in 3D navigation (please see Supplementary Note 7). Hence, arranging the spatial cell bands (excluding OSC band) in the descending order of their PC variance goes as: place cells, border cells, grid cells, and plane cells. This ordering in the eigen spectrum shows correlation with the spatial cell distribution from the LAHN (Fig. 8c). This shows a close relation with the spatial map formation and the PCA-like learning rule.

## Discussion

We present here a hierarchical network model for the formation of a variety of spatial cells in the 3D space which captures the empirical results of 3D spatial cells reported to date^17,18,56^. It further accounts for the puzzling anisotropy of grid-cell coding as reported in rodents by revealing the relationship between the anisotropy and the range of pitch values of the animal’s trajectory. While 3D spatial cell types such as grid cells and border cells have yet to be reported in an animal navigating in the complete 3D space, the model further makes explicit predictions about their expected properties: first, on the spatially periodic representations in 3D space the model contests the emergent periodic structure with the FCC symmetry and shows the possibility of a planar symmetry in 3D space rather than FCC-like lattice structure. This is in partial agreement with the empirical reports that support planar symmetry of the spatial representations in 3D space^51^, notwithstanding the fact that these report emerges from rodents that do not span the complete volumetric space during navigation. This shows the need for a new model organism like bats to study the problem of full 3D navigation^57^. The model also predicts the existence of 3D border cells by showing the emergence of such cells whose 3D rate maps show bias towards any one or two borders (walls) of the box. Importantly, the model predicts the existence of novel spatial cell types, unreported in experimental literature. Apart from the border cells, the model also shows the emergence of neurons that code for a lower-dimensional subspace like a plane (plane cells) or more than one plane (stack cells). The aforementioned results are obtained using anti-Hebbian network, in which the afferent and lateral connections are trained by Hebbian and Stent rules, respectively. These learning rules qualify the biological plausibility of the network and also strengthen the model predictions. To our knowledge, this is the first model to explain the formation of all 3D spatial cells at a systems level.

An extensive empirical study on the navigational behavior of the Egyptian fruit bats resulted in the discovery of 3D place cells in their dorsal hippocampus^18,58^. This extends the implications of cognitive spatial maps onto navigation in higher-dimensional space. 3D place cells exhibit volumetric firing fields on their 3D flight trajectory and cover the entire space uniformly. Around 33% of LAHN neurons in the model show the emergence of localized firing fields akin to the aforementioned empirically reported 3D place cells (Figs. 2b–e and 8c). It is evident from Fig. 2a that the model takes 175,000 iterations to form a localized place-cell activity and this corresponds to ~30 min time period (one iteration takes 0.01 s). This time corresponds to the timescales of empirical experiments conducted in flying bats where 3D spatial firing fields have been reported^18^. Isotropic coding is an important feature that singles out 3D place cells of bats from their rodent counterpart^18^. Place neurons from the model also exhibit isotropic 3D spatial encoding which is evident from the distribution of the elongation index obtained from a fitted ellipsoid (Fig. 2f). The reason for isotropy has been attributed to the evolutionary pressure to encode and decode the 3D SI in an efficient manner^18^.

Place cells and grid cells are empirically reported to exhibit an intriguing transformation of their representations in rodents from classical localized isotropic place field and hexagonal grid firing fields to stripe-like firing fields as navigation changes from horizontal *XY* to vertical *YZ* plane, respectively^52^. The role of the trajectory statistics of a navigating animal in the manifestation of its neural spatial representation cannot be ruled out. This hypothesis has been reinforced by the simulation results shown here (Figs. 4 and 5). We also corroborated the validity of this hypothesis by simulating the reverse case (i.e., training the network by making the animal move on the vertical wall and then testing the network by making the animal move on the horizontal floor), which essentially gave a similar result (i.e., stripes on vertical wall and grids on the horizontal floor; please see Supplementary Note 6). This result is significant because it is generally believed that the type of a spatial cell depends on the model parameters alone. However, our modeling approach suggests that it is a joint effect of both the model parameters (weights) and the behavior. Hence, we attribute this anisotropic coding schema to the variance in the animal’s direction distribution. Learning rules of the anti-Hebbian network performs PCA-like transformation, that is, projecting the high-dimensional spatial inputs provided by the path integration oscillators to those weight vectors in the direction of maximal variance. Place and grid representations are high-level spatial encoding produced by combining the high variance–high-dimensional sensory information. At smaller values of the s.d. of pitch, we observe an anisotropic transformation in both place cells and grid cells in the model. This suggests that the representations formed by the brain to encode any sensory stimulus may not be a static one, but adapts dynamically to sensory inputs.

Empirical data on full 3D spatial navigation is sparse compared to the enormous 2D navigation empirical data from rodents^1–3,6–9,38,54,59^. The proposed model makes many predictions with respect to 3D spatial representations. Indeed, spatially periodic neurons like grid cells have been observed in crawling bats^20^ and there are also experimental efforts using rodents to study the 3D structure of grid cells^51,52^. However, there are no conclusive results regarding the nature of 3D grid cells. In the proposed model, ~24% of LAHN neurons exhibit spatial periodicity (Fig. 8c). The spatially periodic neurons are analyzed to check for any FCC symmetry, which has been previously predicted as the possible form for the 3D grid structure on the basis of their optimal packing efficiency^37,39^. However, grid neurons in the model apparently do not exhibit FCC symmetry (Fig. 3g). Future work must study conditions for emergence of other forms of 3D grid structure such as HCP structure^39,41,51^, body-centered cubic structure, or columnar structure^41^. Further analysis of rotational symmetry on 2D orthogonal planes (Fig. 3h, i) points to both hexagonal and square planar symmetrical nature of the grid neurons in 3D space, compatible with empirical reports from rodent studies^41,51^. However, it is very unlikely for the brain to choose a non-optimal structure (2D hexagons in 3D space) when there is an option to choose a structure (FCC lattice) that is optimal in terms of packing fraction. The question is what does a network like LAHN do when there is not enough variance/information regarding the 3D component because of highly skewed pitch distribution? In such a case, it may rely upon the very next option, a planar hexagon, which is an optimal structure in the subspace (2D space), which has high variance in the azimuth distribution. An increase in the pitch range could possibly bring a transformation from planar to lattice symmetry. Further analysis along these lines shows that as the animal navigates in 3D space in a way that is less pitch-constrained, the more grid representations tends towards FCC regime (Fig. 6g). FCC structure, as mentioned in the previous modeling studies^37,39,40^, appears to offer a highly efficient packing ratio and hence possibly encodes the 3D volumetric space more efficiently. However, the current simulation result shows the possibility for the dependence of the lattice grid structure formation on the trajectory statistics of the animal. Future experimental work in animals navigating in 3D space while exhibiting adequate trajectory variability will be crucial for addressing this important prediction. The model exhibits another interesting encoding scheme of a lower-dimensional subspace, that is, a 2D plane while the animal performs a complete 3D navigation (Fig. 7e–g). We exclude border cells from this category even though border cells also code for a plane (walls of the box). This kind of lower-dimensional coding has got important functional implications. For instance, a horizontal plane cell could potentially code for the animal’s current altitude. This neuronal prediction could be empirically tested only when the animal engages in flight rather than crawl on the ground.

If we analyze the distribution of the spatial cell types formed from the model, it is evident that place and border cells together comprise 60.53% of the total distribution (Fig. 8c). This is consistent with the empirical reports from rat/bat study where both place cells and border cells are reported from the hippocampal formation. (Note: 3D border cells are not reported from flying bats.) Finally, grid cells that comprise 23.97% of the distribution (in the model) also co-exist with the OSCs in the model, but as per the empirical literature grid cells arise once synapse upstream, that is, in the entorhinal cortex^3,20^. When we analyze the eigen spectrum, the grid-cell band comes after the place-cell and border-cell bands (Fig. 8h). This may point to the possibility that the anatomical hierarchy may be performing computations that extract specific eigen bands. For example, the entorhinal cortex may extract the lower eigen bands that possibly carry grid information, whereas the hippocampus may extract the higher eigen bands, which possibly carry place and border information. Since the border cell comes between the place and the grid bands (Fig. 8g), it is highly likely to see those cells in different structures of the hippocampal formation, which has been reported in rodent studies^7,8^. This kind of band separation across anatomical structures could potentially enhance the computational efficacy of the navigation system since each specific information (like place info, border info, displacement info) is well segregated into different bands and also into different anatomical circuits. A possible analog for this band separation is the frequency modulation principle in communication theory where information is embedded at different bands of frequencies. This allows the different bands to not interfere with each other, thereby increasing the efficacy of the information transfer system^60^. Since the model consists of an abstract hierarchy of neuronal layers, a strictly anatomical interpretation of model components is not feasible at the moment. Also, the extensive literature on the quantitative analysis of the anti-Hebbian network (e.g., with *m* number of neurons) has shown that the network essentially minimizes the reconstruction error (quadratic cost function), which after gradient descent optimization gives rise to optimal transformation matrix whose row space is spanned by the eigenvectors corresponding to the *m* highest eigenvalues of the covariance matrix of the input data^61–63^. This means that using the current architecture of the anti-Hebbian network, it is not possible to make the weight connections converge to a restricted subspace until and unless additional constraints are applied to the aforementioned cost function. This analysis (which may include constrained quadratic optimization) could be exhaustive and will need to be addressed in future studies.

To our knowledge, this is the first network-level modeling effort to explain the formation of empirically reported 3D spatial cells along with the predictions on the other possible kinds of spatial cells. However, there are other possible add-ons to this network model. Here, the model is driven by the readily available direction information (both azimuth and pitch), which are assumed to come from the upstream areas like dorsal presubiclum^19^. This directional information could be extracted from the visual, proprioceptive, and echolocation (in the case of bats) sensory information in a more biological way to produce a toroidal map of HD using self-organization principles. This will further allow study of the influence of these sensory modalities on the 3D spatial cells. What are the possible dynamic configurations that the spatial cells can assume to account for any change in the sensory stimulus? We would like to extend this model to study the goal-directed navigation problem in 3D space where the animal searches for a target location and over the course of time it learns to find the target rapidly (this is a benchmark experimental paradigm used to study the 2D navigation problem under the name Morris water-maze task^64^). This will shed light onto the possible ways in which the 3D spatial cells aid the animal to find its goal location.

## Methods

### Azimuth and pitch direction computation

Given change in the position across all the dimensions *∆x*, ∆*y*, and ∆*z*:

1 $$\theta _{{\mathrm{Az}}}(t) = {\mathrm{tan}}^{ - 1}\left[ {{\mathrm{\Delta }}y(t)/{\mathrm{\Delta }}x(t)} \right],$$

2 $$\theta _{\mathrm{P}}(t) = \tan ^{ - 1}\left[ {{\mathrm{\Delta }}z(t)/\sqrt {{\mathrm{\Delta }}x(t)^2 + {\mathrm{\Delta }}y(t)^2} } \right],$$

where *θ*_Az_ and *θ*_P_ are azimuth and pitch directions, respectively.

### HD response

There are a total of *M* azimuth and pitch neurons divided in 7:3 ratio as per the empirical data^19^. Neurons in the azimuth and pitch are tuned to preferred directions that span 360°. Activity of neurons in each layer is computed as given below:

3 $$\phi _{{\mathrm{Az}}}^i(t) = {\mathrm{cos}}[\theta _{{\mathrm{Az}}}(t) - \theta _{{\mathrm{Az}}}^i],$$

4 $$\phi _{\mathrm{P}}^i(t) = {\mathrm{cos}}[\theta _{\mathrm{P}}(t) - \theta _{\mathrm{P}}^i],$$

where $\phi _{{\mathrm{Az}}}^i$ and $\phi _{\mathrm{P}}^i$ are the activities of the azimuth and pitch neurons, respectively, and $\theta _{{\mathrm{Az}}}^i$ and $\theta _{\mathrm{P}}^i$ are the preferred directions of *i*th azimuth and pitch neurons, respectively.

### Path integration layer

Each neuron in this layer is a phase oscillator whose dynamics is given in Eqs. 5 and 6. This layer has one-to-one connectivity with the preceding direction encoding layer. The direction and speed information is integrated into the phase of the oscillators:

5 $$\begin{array}{*{20}{c}} \bullet \\ {\theta _{{\mathrm{PI}}_{{\mathrm{Az}}}}^i} \end{array} = \omega + \beta s\phi _{{\mathrm{Az}}}^i,$$

6 $$y_{{\mathrm{PI}}_{{\mathrm{Az}}}}^i = {\mathrm{sin}}\left( {\theta _{{\mathrm{PI}}_{{\mathrm{Az}}}}^i} \right),$$

where *ω* is the angular frequency of the oscillator such that *ω* = 2π*f*, where *f* is the base frequency of the oscillator, *β* is the modulation factor, and *s* is the speed of navigation given as:

7 $$s = \left\| {{\mathbf{X(t)}} - {\mathbf{X(t}} - {\mathbf{1)}}} \right\|,$$

8 $${\mathbf{X}} \in {\Bbb R}^3.$$

Similar equations (i.e., Eqs. 5 and 6) hold for the pitch path integration layer too (with the only change that the direction input it receives comes from the pitch direction neurons). Empirically, it is reported that the spectrum of local field potential oscillations in the bat hippocampal area lack *θ* oscillations^18,20,45^. Hence, in the model also we used a very low base frequency (*f* = 0.5 Hz) for the oscillators.

### Response equation of anti-Hebbian network

Oscillatory activity (i.e., the phase variable passed through the sine function) in the azimuth and pitch path integration layer (Eq. 6) serves as the input to the downstream LAHN. Hence, both azimuth and pitch path integration values are concatenated together (concatenation operation is shown by the symbol | in Eq. 10) and passed to the downstream neural LAHN layer. This ensures that LAHN layer is endowed with complete 3D information. The activity of a neuron in the LAHN is given as^46^:

9 $$\gamma _i(t) = \mathop {\sum}\limits_{j = 1}^m {q_{ij}y_{{\mathrm{PI}}}^j(t) + \mathop {\sum}\limits_{k = 1}^n {p_{ik}\gamma _k(t - 1)} },$$

10 $$y_{{\mathrm{PI}}} = [y_{{\mathrm{PI}}_{{\mathrm{Az}}}}|y_{{\mathrm{PI}}_{\mathrm{P}}}],$$

where *γ*_*i*_ is the activity of *i*th LAHN neuron, *q*_*ij*_ is the afferent weight connection from *j*th component of input signal *y*_PI_ to *i*th neuron of LAHN, *p*_*ik*_ is the lateral weight connection from *k*th to *i*th LAHN neuron, *m* is the dimension of the input, and *n* is the total number of neurons in LAHN.

### Neural plasticity rule of anti-Hebbian network

The plasticity rules for the synaptic weight connections of LAHN are as given below:

11 $${\mathrm{\Delta }}q_{ij} = \eta _{\mathrm{F}}\left[ {y_{{\mathrm{PI}}}^j(t)\gamma _i(t) - q_{ij}\gamma _i^2(t)} \right],$$

12 $${\mathrm{\Delta }}p_{ik} = - \eta _{\mathrm{L}}\gamma _i(t)\gamma _k\left( {t - 1} \right),$$

where ∆*q*_*ij*_ is the change in the afferent weight connections (Hebbian rule), ∆*p*_*ij*_ is the change in the lateral weight connections (Stent rule), and *η*_F_ and *η*_L_ are the learning rates for the afferent and lateral weight connections, respectively.

The information required for the unsupervised learning of LAHN neuron is available locally at its synaptic connections (Eqs. 11 and 12) and this makes the network biologically plausible.

While training the anti-Hebbian network, we continuously monitored the change in the afferent and lateral weight connections and the training is stopped if it meets the following criteria:

$${\sum} {\left| {{\mathrm{\Delta }}p} \right|} + {\sum} {\left| {{\mathrm{\Delta }}q} \right|} < T,$$

where ∆*p* is the change in the afferent weight connections, ∆*q* is the change in the lateral weight connections, and | | symbol stands for the absolute operation. If the change in the summation of the absolute of both synaptic connections goes less than a tolerance value *T*, the training is stopped since it assures the convergence of both weight connections.

Empirically, a typical behavioral session of the bat takes ~30 min duration (31.8 ± 6.9 min data obtained from the Supplementary Information of ref. ^18^). In the proposed modeling study, the total sample size of a simulated 3D trajectory for training is 175,000 samples and the integration time constant (d*t*) used for the generation of this trajectory is 0.01 s (see Supplementary Note 1 for the generation of the random 3D trajectory). Hence, the total time taken for training during the active 3D navigation of the virtual animal is ~29.2 min, which is on par with the empirical data^18^.

### 3D firing rate map formation

3D volume of the physical space is binned into 41 × 41 × 41 voxels. Neuronal activity is assigned to the respective voxel depending on the firing field position. After this, the rate map is smoothed by a 3D Gaussian filter of *σ* = 3.

### 3D autocorrelation map formation

Autocorrelation map is mainly computed to analyze the spatial periodicity of the neural activity by computing the respective gridness scores. Autocorrelation map, **r**, is computed as follows:

13 $$ {\mathbf{r}}\left( {\tau _x,\tau _y,\tau _z} \right) \\ 	\!\!= {\textstyle{{M\mathop {\sum}\limits_{x,y,z} {{\mathbf{\lambda }}\left( {x,y,z} \right){\mathbf{\lambda }}\left( {x - \tau _x,y - \tau _y,z - \tau _z} \right) - \mathop {\sum}\limits_{x,y,z} {{\mathbf{\lambda }}\left( {x,y,z} \right)\mathop {\sum}\limits_{x,y,z} {{\mathbf{\lambda }}\left( {x - \tau _x,y - \tau _y,z - \tau _z} \right)} } } } \over {\sqrt {\left[ {M\mathop {\sum}\limits_{x,y,z} {{\mathbf{\lambda }}(x,y,z)^2 - \left[ {\mathop {\sum}\limits_{x,y,z} {{\mathbf{\lambda }}(x,y,z)} } \right]^2} } \right]\left[ {M\mathop {\sum}\limits_{x,y,z} {{\mathbf{\lambda }}\left( {x - \tau _x,y - \tau _y,z - \tau _z} \right)^2 - \left[ {{\mathbf{\lambda }}(x - \tau _x,y - \tau _y,z - \tau _z)} \right]^2} } \right]} }}},$$

where **λ**(*x*, *y*, *z*) is the firing rate at (*x*, *y*, *z*) location of the rate map, *M* is the total number of voxels in the rate map, and *τ*_*x*_*, τ*_*y*_, and *τ*_*z*_ correspond to *x*, *y*, and *z* coordinate spatial lags.

### Fitting a plane to the firing field

Since the firing field in this case has three dimensions, multiple regression is done to fit a plane to the firing field data. The model is as follows^65^:

$$\hat y = \alpha _0 + \alpha _1x_1 + \alpha _2x_2,$$

where *ŷ* is the variable that has to be predicted, *α*'s are the regression coefficients that has to be determined, and *x*_1_ and *x*_2_ are the regressors.

Regressors are computed by minimizing the sum of squares of the residuals (SSR) such as:

14 $${\mathrm{SSR}} = \mathop {\sum}\limits_i {\left( {y_i - \hat y_i} \right)^2},$$

where *y*_*i*_ is the actual value and *ŷ*_*i*_ is the predicted value from the multiple regression model.

### Fitting an ellipsoid to the place-cell firing field

Ellipsoid is fitted to the firing field data of a place-cell by minimizing the following cost function:

15 $${\mathrm{Min}}\left| {\frac{{(x - c_x)^2}}{{r_x^2}} + \frac{{(y - c_y)^2}}{{r_y^2}} + \frac{{(z - c_z)^2}}{{r_z^2}} - 1} \right|,$$

where *c*_*x*_, *c*_*y*_, and *c*_*z*_ are the coordinates of the center of the ellipsoid in the 3D Cartesian coordinate system, and *r*_*x*_, *r*_*y*_, and *r*_*z*_ are the semi-axis lengths of the ellipsoid.

### Computation of spatial descriptors

SI index is used to classify if a neuron carries any spatially relevant information or not^18^. It is computed from the neuron’s firing rate map as given below:

16 $${\mathrm{SI}} = \mathop {\sum}\limits_i {p_i\frac{{{\mathbf{\lambda }}_i}}{{\mathbf{\lambda }}}{\mathrm{log}}_2\left( {\frac{{{\mathbf{\lambda }}_i}}{{\mathbf{\lambda }}}} \right)},$$

where *p*_*i*_ is the probability for the animal being in the *i*th voxel. This can be computed as the ratio of the number of times the animal visited that voxel to the total time of its flight. **λ**_*i*_ is the firing rate in the *i*th voxel. **λ** is the mean firing rate across the rate map. SI is expressed as bits per spike.

Gridness score is used to analyze the spatially periodic grid activity of a neuron. We adopted the standard methodology for the gridness score computation as mentioned in ref. ^3^. From the 2D autocorrelogram of the neuron (this can be the projections of a 3D autocorrelogram on a plane), the local peaks near to the central peak of the autocorrelogram are cropped out and the central peak is further masked. Following this, 60° and 90° rotational symmetry of the autocorrelograms are further analyzed to quantify the HGS and SGS gridness score of a neuron, respectively. The relevant equations are as shown below:

17 $${\mathrm{HGS}} 	= \,{\mathrm{min}}\left[ {{\mathrm{cor}}\left( {{\mathbf{r}},{\mathbf{r}}^{60^0}} \right),{\mathrm{cor}}\left( {{\mathbf{r}},{\mathbf{r}}^{120^0}} \right)} \right] \\ 	- {\mathrm{max}}\left[ {{\mathrm{cor}}\left( {{\mathbf{r}},{\mathbf{r}}^{30^0}} \right),{\mathrm{cor}}\left( {{\mathbf{r}},{\mathbf{r}}^{90^0}} \right),{\mathrm{cor}}\left( {{\mathbf{r}},{\mathbf{r}}^{150^0}} \right)} \right],$$

SGS is computed as follows^21^:

18 $${\mathrm{SGS}} = {\mathrm{cor}}\left( {{\mathbf{r}},{\mathbf{r}}^{90^0}} \right) - {\mathrm{max}}\left[ {{\mathrm{cor}}\left( {{\mathbf{r}},{\mathbf{r}}^{45^0}} \right),{\mathrm{cor}}\left( {{\mathbf{r}},{\mathbf{r}}^{135^0}} \right)} \right],$$

where **r**_*θ*_ is the autocorrelation map rotated by *θ*°.

BS is used to analyze the border activity of a neuron. To compute BS, two quantities are assessed such as: the maximal extent of a single field on any wall (*C*_M_) and the mean firing distance (*d*_M_). *d*_M_ is computed as the average distance of each bin in the firing rate map to the nearest wall, weighted by the firing rate activity in that bin. BS is then computed as follows^7,54^:

19 $${\mathrm{BS}} = \frac{{C_{\mathrm{M}} - d_{\mathrm{M}}}}{{C_{\mathrm{M}} + d_{\mathrm{M}}}}.$$

BS ranges between −1 and 1, where −1 represents a central firing and +1 represents a firing field that is perfectly aligned with a border.

PI is used to analyze the planeness of a neuron. This is obtained by fitting a plane to the 3D firing field, and the goodness-of-fit *R*^2^ value is considered as the PI score. *R*^2^ value is the coefficient of determination whose value ranges between 0 and 1. It is defined as the proportion of the variance in the dependent variable that can be explained using the independent variable^65^. *R*^2^ value can be estimated as follows:

20 $$R^2 = 1 - \frac{{{\mathrm{SSR}}}}{{{\mathrm{SST}}}},$$

where SSR is the sum of squared residuals as mentioned above.

SST is the total sum of squares which gives the information on the total variance of the data around its mean. It is given as follows:

$${\mathrm{SST}} = \mathop {\sum}\limits_i {\left( {y_i - \bar y} \right)^2},$$

where *y̅* is the mean of the data to be predicted. Fitting of Gaussian distribution on the histogram of data is done using histfit function in MATLAB.

All simulations are done in MATLAB R2016a. Please see Table 1 for the parameter values.

**Table 1:** Parameter values

Parameter	Value
Number of LAHN neurons	50
*f*	0.5 Hz
*N*_Az_	70
*N*_P_	30
*β*	2
Spatial information threshold	1.0609
Hexagonal gridness score threshold	0.1686
Square gridness score threshold	0.1952
Border score threshold	0.5228
Plane index threshold	0.7528
*η*_F_	0.01
*η*_L_	0.01
d*t*	0.01 s

## Electronic supplementary material

Supplementary Information

## Data Availability

All the relevant data and code are available from the corresponding author upon reasonable request.

## References

[1] O’Keefe J, Dostrovsky J (1971). The hippocampus as a spatial map. Preliminary evidence from unit activity in the freely-moving rat. Brain Res..

[2] Moser EI (2014). Grid cells and cortical representation. Nat. Rev. Neurosci..

[3] Hafting T, Fyhn M, Molden S, Moser MB, Moser EI (2005). Microstructure of a spatial map in the entorhinal cortex. Nature.

[4] Taube JS, Muller RU, Ranck JB (1990). Head-direction cells recorded from the postsubiculum in freely moving rats. I. Description and quantitative analysis. J. Neurosci..

[5] Taube JS, Muller RU, Ranck JB (1990). Head-direction cells recorded from the postsubiculum in freely moving rats. II. Effects of environmental manipulations. J. Neurosci..

[6] Taube JS, Bassett JP (2003). Persistent neural activity in head direction cells. Cereb. Cortex.

[7] Solstad T, Boccara CN, Kropff E, Moser MB, Moser EI (2008). Representation of geometric borders in the entorhinal cortex. Science.

[8] Lever C, Burton S, Jeewajee A, O’Keefe J, Burgess N (2009). Boundary vector cells in the subiculum of the hippocampal formation. J. Neurosci..

[9] Bjerknes TL, Moser EI, Moser MB (2014). Representation of geometric borders in the developing rat. Neuron.

[10] Knierim JJ, McNaughton BL (2001). Hippocampal place-cell firing during movement in three-dimensional space. J. Neurophysiol..

[11] Stackman RW, Taube JS (1998). Firing properties of rat lateral mammillary single units: head direction, head pitch, and angular head velocity. J. Neurosci..

[12] Bassett JP, Taube JS (2001). Neural correlates for angular head velocity in the rat dorsal tegmental nucleus. J. Neurosci..

[13] Calton JL, Taube JS (2005). Degradation of head direction cell activity during inverted locomotion. J. Neurosci..

[14] Stackman RW, Tullman ML, Taube JS (2000). Maintenance of rat head direction cell firing during locomotion in the vertical plane. J. Neurophysiol..

[15] Ulanovsky N, Moss CF (2007). Hippocampal cellular and network activity in freely moving echolocating bats. Nat. Neurosci..

[16] Ulanovsky N (2011). Neuroscience: how is three-dimensional space encoded in the brain?. Curr. Biol..

[17] Yartsev MMJS (2013). Space bats: multidimensional spatial representation in the bat. Science.

[18] Yartsev MM, Ulanovsky N (2013). Representation of three-dimensional space in the hippocampus of flying bats. Science.

[19] Finkelstein A (2015). Three-dimensional head-direction coding in the bat brain. Nature.

[20] Yartsev MM, Witter MP, Ulanovsky N (2011). Grid cells without theta oscillations in the entorhinal cortex of bats. Nature.

[21] Soman Karthik, Muralidharan Vignesh, Chakravarthy V. Srinivasa (2018). A Model of Multisensory Integration and Its Influence on Hippocampal Spatial Cell Responses. IEEE Transactions on Cognitive and Developmental Systems.

[22] Burgess N, Barry C, O’Keefe J (2007). An oscillatory interference model of grid cell firing. Hippocampus.

[23] Burak Y, Fiete IR (2009). Accurate path integration in continuous attractor network models of grid cells. PLoS Comput. Biol..

[24] Fuhs MC, Touretzky DS (2006). A spin glass model of path integration in rat medial entorhinal cortex. J. Neurosci..

[25] Zilli EA, Hasselmo ME (2010). Coupled noisy spiking neurons as velocity-controlled oscillators in a model of grid cell spatial firing. J. Neurosci..

[26] Widloski J, Fiete IR (2014). A model of grid cell development through spatial exploration and spike time-dependent plasticity. Neuron.

[27] Bush D, Burgess N (2014). A hybrid oscillatory interference/continuous attractor network model of grid cell firing. J. Neurosci..

[28] Burgess N, O’Keefe J (2011). Models of place and grid cell firing and theta rhythmicity. Curr. Opin. Neurobiol..

[29] Kropff E, Treves A (2008). The emergence of grid cells: Intelligent design or just adaptation?. Hippocampus.

[30] Gaussier P (2007). A model of grid cells involving extra hippocampal path integration, and the hippocampal loop. J. Integr. Neurosci..

[31] Mhatre H, Gorchetchnikov A, Grossberg S (2012). Grid cell hexagonal patterns formed by fast self‐organized learning within entorhinal cortex. Hippocampus.

[32] Soman K, Muralidharan V, Chakravarthy VS (2018). A unified hierarchical oscillatory network model of head direction cells, spatially periodic cells, and place cells. Eur. J. Neurosci..

[33] Bicanski A, Burgess N (2016). Environmental anchoring of head direction in a computational model of retrosplenial cortex. J. Neurosci..

[34] Blair HT, Gupta K, Zhang K (2008). Conversion of a phase‐to a rate‐coded position signal by a three‐stage model of theta cells, grid cells, and place cells. Hippocampus.

[35] Guanella A, Kiper D, Verschure P (2007). A model of grid cells based on a twisted torus topology. Int. J. Neural Syst..

[36] Jeffery K, Burgess N (2006). The boundary vector cell model of place-cell firing and spatial memory. Rev. Neurosci..

[37] Mathis A, Stemmler MB, Herz AV (2015). Probable nature of higher-dimensional symmetries underlying mammalian grid-cell activity patterns. eLife.

[38] Bonnevie T (2013). Grid cells require excitatory drive from the hippocampus. Nat. Neurosci..

[39] Stella F, Treves A (2015). The self-organization of grid cells in 3D. eLife.

[40] Horiuchi TK, Moss CF (2015). Grid cells in 3-D: reconciling data and models. Hippocampus.

[41] Jeffery, K. J., Wilson, J. J., Casali, G. & Hayman, R. M. Neural encoding of large-scale three-dimensional space—properties and constraints. *Front. Psychol.* **6**, 927 (2015). 10.3389/fpsyg.2015.00927 PMC4501222 26236246

[42] Laurens J, Angelaki DE (2018). The brain compass: a perspective on how self-motion updates the head direction cell attractor. Neuron.

[43] Laurens J, Kim B, Dickman JD, Angelaki DE (2016). Gravity orientation tuning in macaque anterior thalamus. Nat. Neurosci..

[44] Page HJI, Wilson JJ, Jeffery KJ (2018). A dual-axis rotation rule for updating the head direction cell reference frame during movement in three dimensions. J. Neurophysiol..

[45] Heys JG, MacLeod KM, Moss CF, Hasselmo ME (2013). Bat and rat neurons differ in theta-frequency resonance despite similar coding of space. Science.

[46] Földiak P (1990). Forming sparse representations by local anti-Hebbian learning. Biol. Cybern..

[47] Stent GS (1973). A physiological mechanism for Hebb’s postulate of learning. Proc. Natl. Acad. Sci. USA.

[48] Sanger TD (1989). Optimal unsupervised learning in a single-layer linear feedforward neural network. Neural Netw..

[49] Oja E (1982). A simplified neuron model as a principal component analyzer. J. Math. Biol..

[50] Omer DB, Maimon SR, Las L, Ulanovsky N (2018). Social place-cells in the bat hippocampus. Science.

[51] Hayman, R. M. A., Casali, G., Wilson, J. J. & Jeffery, K. J. Grid cells on steeply sloping terrain: evidence for planar rather than volumetric encoding. *Front. Psychol*. **6**, 925 (2015). 10.3389/fpsyg.2015.00925 PMC4502341 26236245

[52] Hayman R, Verriotis MA, Jovalekic A, Fenton AA, Jeffery KJ (2011). Anisotropic encoding of three-dimensional space by place cells and grid cells. Nat. Neurosci..

[53] Conway JH, Sloane NJA (2013). Sphere Packings, Lattices and Groups.

[54] Diehl GW, Hon OJ, Leutgeb S, Leutgeb JK (2017). Grid and nongrid cells in medial entorhinal cortex represent spatial location and environmental features with complementary coding schemes. Neuron.

[55] Wold S, Esbensen K, Geladi P (1987). Principal component analysis. Chemom. Intell. Lab. Syst..

[56] Finkelstein A, Las L, Ulanovsky N (2016). 3-D maps and compasses in the brain. Annu. Rev. Neurosci..

[57] Yartsev MMJS (2017). The emperor’s new wardrobe: rebalancing diversity of animal models in neuroscience research. Science.

[58] Geva-Sagiv M, Romani S, Las L, Ulanovsky N (2016). Hippocampal global remapping for different sensory modalities in flying bats. Nat. Neurosci..

[59] Barry C, Hayman R, Burgess N, Jeffery KJ (2007). Experience-dependent rescaling of entorhinal grids. Nat. Neurosci..

[60] Haykin SS, Moher M, Song T (1989). An Introduction to Analog and Digital Communications.

[61] Kung SY, Diamantaras KI, Taur JS (1994). Adaptive principal component extraction (Apex) and applications. IEEE Trans. Signal Process..

[62] Pehlevan C, Hu T, Chklovskii DB (2015). A hebbian/anti-hebbian neural network for linear subspace learning: a derivation from multidimensional scaling of streaming data. Neural Comput..

[63] Yang B (1995). Projection approximation subspace tracking. IEEE Trans. Signal Process..

[64] Morris R (1984). Developments of a water-maze procedure for studying spatial learning in the rat. J. Neurosci. Methods.

[65] Tabachnick BG, Fidell LS (2007). Using Multivariate Statistics.

