# Supplementary material for: A hierarchical anti-Hebbian network model for the formation of spatial cells in three-dimensional space
Source: Nat Commun. 2018 Oct 2;9:4046. doi: 10.1038/s41467-018-06441-5 (PMC6168468; doi:10.1038/s41467-018-06441-5)
Supplement: Supplementary file 1 — Supplementary Information [file 41467_2018_6441_MOESM1_ESM.pdf]

Supplementary Materials for

# A Hierarchical Anti-Hebbian Network Model for the Formation of Spatial Cells in Three-Dimensional Space

Karthik Soman[1,2], Srinivasa Chakravarthy[1], Michael M. Yartsev[2]

[1]Bhupat and Jyoti Mehta School of Biosciences, Department of Biotechnology, Indian Institute of Technology Madras, Chennai, Tamilnadu, India - 600036.

[2]Department of Bioengineering and the Helen Wills Neuroscience Institute, University of California–Berkeley, Berkeley, CA 94708, USA.

This supplementary material includes the following sections:

Supplementary Note 1: Generation of 3D trajectory

Supplementary Note 2: Procedure for the shuffling analysis

a. Elongation index
b. Grid score
c. Border score
d. Plane cell index
e. Spatial information index

Supplementary Note 3: Generation of transection planes in FCC analysis

Supplementary Note 4: Quantitative analysis of positional encoding by the temporal oscillations

a. Generation of different spatial representations
b. Dependence on initial position of the animal

Supplementary Note 5: Frequency variation analysis

Supplementary Note 6: Simulation result after training the animal on the vertical plane

Supplementary Note 7: Additional spatial representations from the model

Supplementary References

Number of figures in the Supplementary material - 15

**Supplementary Note 1: Generation of 3D trajectory**

The 3D Cartesian coordinates i.e. *x*, *y* and *z* coordinates of the 3D trajectory are generated using the following dynamical equations:

Dynamics of the Cartesian coordinates are as follows:

$$\dot{x}(t) = \sigma(t)\cos[\theta_{\mathrm{Az}}(t)]\sin[\theta_P(t)]$$

$$\dot{y}(t) = \sigma(t)\sin[\theta_{\mathrm{Az}}(t)]\sin[\theta_P(t)]$$

$$\dot{z}(t) = \sigma(t)\cos[\theta_P(t)]$$

$$\sigma(t) = \| \mathbf{X}_{\mathrm{pos}}(t) - \mathbf{X}_{\mathrm{wall}} \|$$

$\sigma(t)$ makes sure that the virtual animal does not cross the borders of the box. $X_{\mathrm{pos}}$ is the current position of the animal and $X_{\mathrm{wall}}$ is the coordinate of a point on the wall that would intersect with the 3D direction vector of the animal.

Pitch angle is sampled from a Normal distribution parameterized by the mean ($\mu$) and the variance ($\sigma^2$).

$$\theta_P(t) \sim \mathrm{N}(\mu, \sigma^2)$$

Further an upper bound is given to the change in the pitch angle to make sure that the animal does not take a sharp pitch.

Azimuth angle is sampled from a Uniform distribution that spans the entire 360° angular space. In the case of azimuth angle an additional constraint is also included i.e. if the animal is moving closer to any border of the box (distance value is less than a threshold distance to the wall), additional dynamics is incorporated for the azimuth angle (rather than sampling from the Uniform distribution) as shown below.

If $\sigma(t) < \mathrm{wall}_{\mathrm{thresh}}$

$$\dot{\theta}_{\mathrm{Az}} = \frac{\gamma_{\mathrm{Az}}}{\sigma(t) + \alpha}$$

else $\theta_{\mathrm{Az}}(t) \sim U(0^0, 360^0)$

end

$\gamma_{\mathrm{Az}}$ is a parameter that scales the Azimuth dynamics.

$\alpha \ll 1$ to ensure that the fraction does not go to infinity when $\sigma(t)$ tends to zero. Also, an upper bound is given to the change in the azimuth angle to make sure that the animal does not take a sharp azimuth.

**Supplementary Note 2: Procedure for the shuffling analysis**

Shuffling analysis was conducted to choose an optimal threshold value for the following spatial descriptors:

a. Elongation index

We followed a procedure similar to the one described in [1] to determine whether the elongation index of a place field is statistically different from that expected from a spherical shape. First, for each field, we defined a perfect sphere whose center matched the center of the fitted ellipsoid and whose diameter equaled the geometric mean of the ellipsoid's three full-axes so that the volume of the sphere was identical to the volume of the original ellipsoid. Second, the neural activity within each field was randomly distributed over the flight-trajectories that passed inside the sphere. Then the shuffled place-field was calculated, and its elongation index was computed in the same way as described in the manuscript. This shuffling procedure was repeated 1000 times for each place-field. 95$^{th}$ percentile of this shuffled distribution is taken as the threshold for the elongation index. The place cell whose elongation index value is below this computed threshold is considered to be an isotropic place cell. Supplementary figure 1 shows the computed distribution. 95$^{th}$ percentile value is shown as the red line and the actual value is given on the top of the distribution (1.3797).

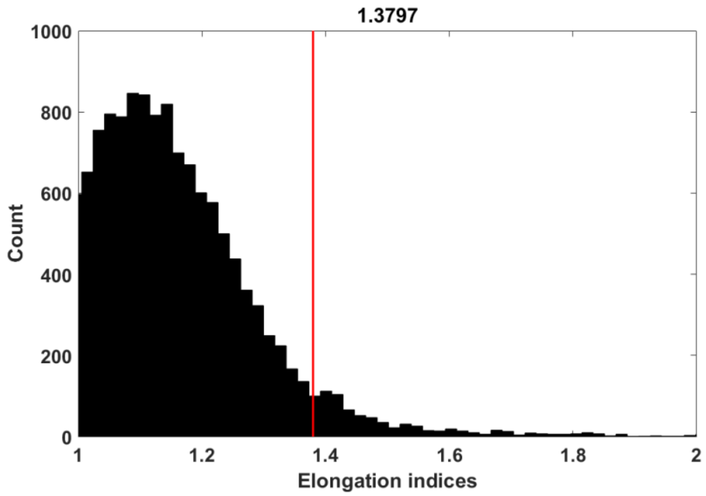


Supplementary Figure 1: Distribution of elongation indices after shuffling process. Red line indicates the 95$^{th}$ percentile of the distribution and the corresponding value is given at the top of the figure.

b. Grid score

To assess the significance of the gridness score obtained for each simulated neuron, we compared the actual gridness score value to the gridness score values computed for a set of shuffled neural activity, as follows:

The shuffled distribution was generated by randomly distributing the neural activity across the simulated flight trajectory. This shuffling procedure was repeated 1000 times for each neuron (50 neurons in the anti-Hebbian network). For each repetition, the firing-rate maps were computed in the same manner as described in the manuscript (in the methods section), and the gridness score value was then computed (i.e.

by projecting the shuffled 3D rate map on the three major planes such as XY, XZ and YZ and then computing the gridness score on each plane. The maximum value out of the three is considered as the gridness score for that neuron). Neurons whose gridness score exceeded the upper boundary of their shuffled-distribution (95$^{th}$ percentile), i.e. which showed a gridness score higher than would be expected by chance, were defined as hexagonal grid cells. Apart from the hexagonal grid, we also analyzed the shuffled distribution for the square gridness score. The distributions are shown below as Supplementary Figures 2 and 3 and the threshold value (95$^{th}$ percentile of the distribution) is marked as red line and written at the top of each distribution.

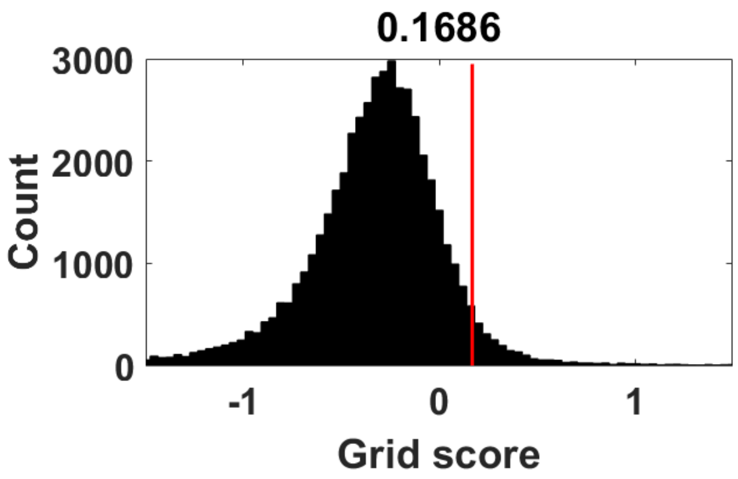


Supplementary Figure 2: Distribution of hexagonal gridness score after shuffling process. Red line indicates the 95$^{th}$ percentile of the distribution and the corresponding value is given at the top of the figure.

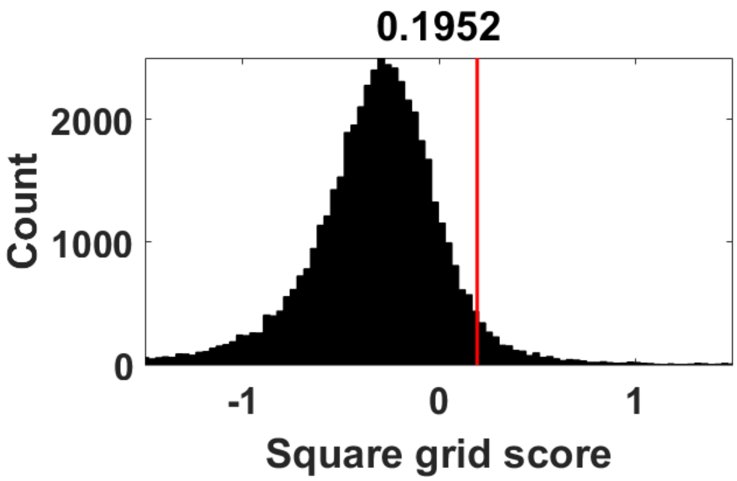


Supplementary Figure 3: Distribution of square gridness score after shuffling process. Red line indicates the 95$^{th}$ percentile of the distribution and the corresponding value is given at the top of the figure.

c. Border score

The border score of a neuron is analyzed using the following steps:

1. Project 3D volumetric rate map onto 3 major planes (XY, YZ and XZ) and compute the 2D border scores for each plane.
2. Arrange the three border scores in the descending order.
3. If the first two border scores in the list are > border threshold value, it qualifies as a 3D border cell and its border score will be the average of the top two border scores in the list.

To compute the border threshold, we conducted the shuffling analysis where the activity of each neuron is shuffled as explained previously and the border score is computed for each shuffled rate map using the aforementioned three steps. This shuffling procedure generates a distribution of the border scores and the threshold is computed as the $95^{th}$ percentile of this distribution as shown in Supplementary Figure 4.

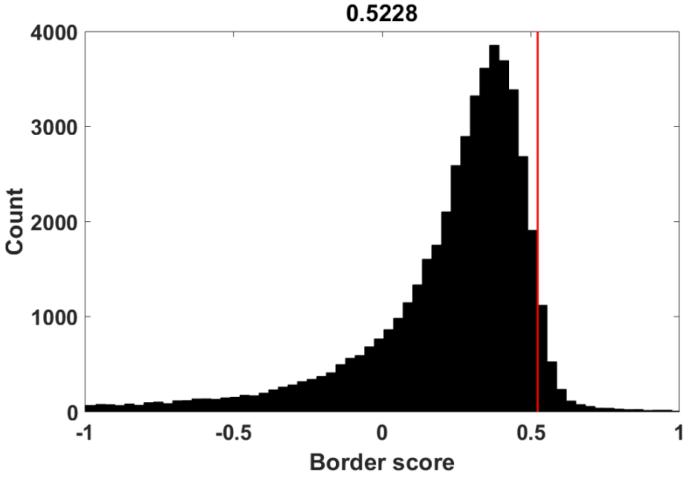


Supplementary Figure 4: Distribution of border score after shuffling process. Red line indicates the $95^{th}$ percentile of the distribution and the corresponding value is given at the top of the figure.

d. Plane cell index

To check the plane information carried by the neuron, a plane is fitted to the 3D firing field of the neuron and the goodness of fit ($R^2$ value) is considered as the plane index of that neuron as mentioned in the methods section of the manuscript. A shuffling of the neural activity (as done in the previous cases mentioned above) resulted in the following distribution (Supplementary Figure 5) and the $95^{th}$ percentile of the distribution is considered as the plane index threshold. A neuron qualifies as a plane cell if its plane index crosses the plane threshold value and also if its border score value is less than the border threshold (or else a border cell will be classified as a plane cell).

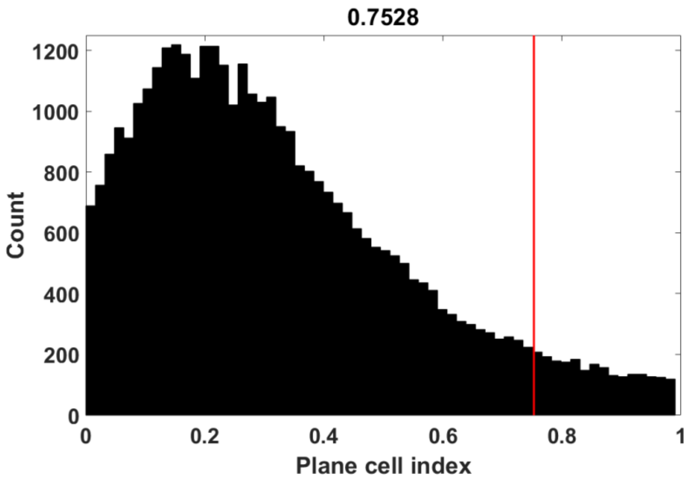


Supplementary Figure 5: Distribution of plane index after shuffling process. Red line indicates the 95th percentile of the distribution and the corresponding value is given at the top of the figure.

e. Spatial information index

Spatial information (SI) quantifies the amount of information that the neuron carries about the space. SI is computed from the rate map of a neuron using the method adopted from [2]. Please see Eqn 16 in the manuscript for the closed form expression of the SI. To compute the threshold for the SI, we did the shuffling analysis in the similar way that was done for the other spatial descriptors and the 95th percentile of the distribution (Supplementary Figure 6) was considered as the SI threshold. Any neuron whose SI index is greater than this threshold is considered as a spatial cell. Specifically to classify the place cells we considered all the neurons that come under the category of grid, border and plane cells based on their respective descriptors. Since place cell has a localized firing activity, theoretically it should have more SI compared to the other spatial cells. Hence, place cell SI threshold is computed as the maximum of the SI of all these spatial cells (which has come to 2.6).

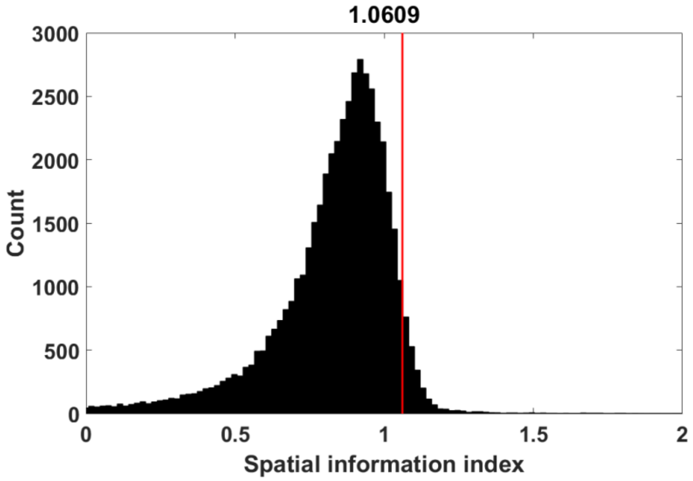


Supplementary Figure 6: Distribution of spatial information index after shuffling process. Red line indicates the 95th percentile of the distribution and the corresponding value is given at the top of the figure.

**Supplementary Note 3: Generation of transection planes in FCC analysis**

Transection planes are generated using the following steps:

1. Form an array of 3D vectors that serve as normal vectors to different transection planes.
   A single 3D vector is parameterized using the azimuth ($\theta_{\text{Az}}$) and pitch angle ($\theta_P$) as shown in the following spherical coordinate system equation:

   $$\vec{N} = [\cos(\theta_{\text{Az}})\sin(\theta_P)\ \ \sin(\theta_{\text{Az}})\sin(\theta_P)\ \ \cos(\theta_P)]$$

   Using the normal vectors, we use extract slice function in MATLAB to transect the 3D autocorrelation map. The gridness score of each transected 2D slice is computed and the slice with the maximum gridness score is selected as the reference plane.

2. Three other planes are computed as follows: [as in [3]]

   First plane: Computing the plane essentially means computing the normal vector that defines the plane. To find the normal vector which is at 72° with the reference plane, we adopted a brute force method in which we scanned the complete 360 Azimuth x 360 Pitch combinations (with a resolution of 1° for each, hence a total of 129600 angular combinations) and selected that azimuth-pitch combination which essentially gave a vector that is at 72° to the normal vector of the reference plane (There can be more than one vector which is at 72° to the reference plane and hence we selected that vector whose corresponding plane has the highest gridness score).

   Second plane: We adopted the same method as mentioned for the first plane, but here we found the normal vector which is at 72° to the normal vector of the first plane (Here as well, there can be more than one vector which is at 72° to the first plane and hence we selected that vector whose corresponding plane has the highest gridness score).

   Third plane: Since the third plane needs to be at 72° to both the first and the second plane we needed to find the relevant $\theta_{Az}$ and $\theta_P$ for the third plane. Hence, here also we approached the same brute force method, that is by scanning over all the possible azimuth and pitch angles as mentioned above and find that combination of azimuth and pitch angle that essentially gives rise to a plane which is ~72° with the first and the second plane (Here again, there can be more than one vector which is at 72° to the first and second planes and hence we selected that vector whose corresponding plane has the highest gridness score).

   Supplementary Figure 7 shows three such transection planes obtained from the aforementioned methods passing through an FCC lattice structure (3 planes are shown in 3 different colors such as red, green and blue respectively).

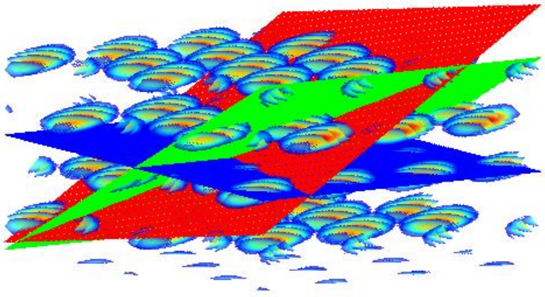

Supplementary Figure 7: Three planes at 72° that transect the 3D FCC lattice structure. Planes are shown in 3 different colors such as red, green and blue respectively.

**Supplementary Note 4: Quantitative analysis of positional encoding by the temporal oscillations**

A 3D vector, **v**, in the spherical coordinate system is represented as follows:

$$\vec{\mathbf{v}} = \begin{bmatrix} \cos(\theta_{\mathrm{Az}})\cos(\theta_p) \\ \sin(\theta_{\mathrm{Az}})\cos(\theta_p) \\ \sin(\theta_p) \end{bmatrix}$$

$$\cos[\theta_{\mathrm{Az}}(t)] = \cos[\tan^{-1}(\Delta y / \Delta x)]$$

$$= \frac{\Delta x}{(\Delta x^2 + \Delta y^2)^{1/2}}$$

$$\sin[\theta_{\mathrm{Az}}(t)] = \frac{\Delta y}{(\Delta x^2 + \Delta y^2)^{1/2}}$$

$$\cos[\theta_P(t)] = \cos\left[\tan^{-1}\left(\frac{\Delta z}{(\Delta x^2 + \Delta y^2)^{1/2}}\right)\right]$$

$$= \frac{(\Delta x^2 + \Delta y^2)^{1/2}}{(\Delta x^2 + \Delta y^2 + \Delta z^2)^{1/2}}$$

$$\sin[\theta_P(t)] = \frac{\Delta z}{(\Delta x^2 + \Delta y^2 + \Delta z^2)^{1/2}}$$

From the above expression, the preferred basis vector of $i^{\text{th}}$ Azimuth neuron can be expressed as follows (considering $\theta_P = 0$):

$$\vec{\mathbf{v}}^{i}_{\text{Az}} = \begin{bmatrix} \cos(\theta^{i}_{\text{Az}}) \\ \sin(\theta^{i}_{\text{Az}}) \\ 0 \end{bmatrix}$$

The preferred basis vector of $i^{\text{th}}$ Pitch neuron can be expressed as follow (considering $\theta_{\text{Az}} = 0$):

$$\vec{\mathbf{v}}^{i}_{P} = \begin{bmatrix} \cos(\theta^{i}_{P}) \\ 0 \\ \sin(\theta^{i}_{P}) \end{bmatrix}$$

Considering the phase dynamics of the $i^{\text{th}}$ Azimuth PI layer:

$$\begin{aligned}\dot{\theta}^{i}_{Az} &= \omega + \beta s\, \vec{\mathbf{v}} . \vec{\mathbf{v}}^{i}_{\text{Az}} \\ &= \omega + \beta s \left\{ \cos[\theta_{\text{Az}}(t)]\cos(\theta^{i}_{\text{Az}}) + \sin[\theta_{\text{Az}}(t)]\sin(\theta^{i}_{\text{Az}}) \right\} \\ &= \omega + \beta s \left[ \frac{\Delta x}{(\Delta x^2 + \Delta y^2)^{1/2}} \cos(\theta^{i}_{Az}) + \frac{\Delta y}{(\Delta x^2 + \Delta y^2)^{1/2}} \sin(\theta^{i}_{Az}) \right]\end{aligned}$$

Applying the expression of the speed

$$= \omega + \beta(\Delta x^2 + \Delta y^2 + \Delta z^2)^{1/2} \left[ \frac{\Delta x}{(\Delta x^2 + \Delta y^2)^{1/2}} \cos(\theta^{i}_{\text{Az}}) + \frac{\Delta y}{(\Delta x^2 + \Delta y^2)^{1/2}} \sin(\theta^{i}_{\text{Az}}) \right]$$

---------------(1)

Since in the simulation the variance in the $z$-direction is less, $\Delta z$ is a small quantity and $\Delta z^2 \rightarrow 0$, hence Supplementary equation (1) can be approximated to:

$$\dot{\theta}^{i}_{\text{Az}} = \omega + \beta \left[ \Delta x \cos(\theta^{i}_{\text{Az}}) + \Delta y \sin(\theta^{i}_{\text{Az}}) \right] \qquad \text{---------------(2)}$$

Considering the phase dynamics of the $i^{\text{th}}$ Pitch PI layer:

$$\begin{aligned}\dot{\theta}^{i}_{P} &= \omega + \beta s\, \vec{\mathbf{v}} . \vec{\mathbf{v}}^{i}_{P} \\ &= \omega + \beta s \left\{ \cos[\theta_P(t)]\cos(\theta^{i}_{P}) + \sin[\theta_P(t)]\sin(\theta^{i}_{P}) \right\} \\ &= \omega + \beta s \left[ \frac{(\Delta x^2 + \Delta y^2)^{1/2}}{(\Delta x^2 + \Delta y^2 + \Delta z^2)^{1/2}} \cos(\theta^{i}_{P}) + \frac{\Delta z}{(\Delta x^2 + \Delta y^2 + \Delta z^2)^{1/2}} \sin(\theta^{i}_{P}) \right]\end{aligned}$$

Applying the expression of the speed

$$= \omega + \beta(\Delta x^2 + \Delta y^2 + \Delta z^2)^{1/2}\left[\frac{(\Delta x^2 + \Delta y^2)^{1/2}}{(\Delta x^2 + \Delta y^2 + \Delta z^2)^{1/2}}\cos(\theta_P^i) + \frac{\Delta z}{(\Delta x^2 + \Delta y^2 + \Delta z^2)^{1/2}}\sin(\theta_P^i)\right]$$

$$= \omega + \beta\left[(\Delta x^2 + \Delta y^2)^{1/2}\cos(\theta_P^i) + \Delta z\sin(\theta_P^i)\right]$$

---------------------- (3)

Hence, Supplementary equations (2) and (3) describe the phase dynamics of the azimuth and the pitch path integration oscillators.

a. Generation of different spatial representations

It is evident from Supplementary equations (2) and (3) that Azimuth PI layer carries the information about the *x*-*y* position of the animal and the Pitch PI layer encodes the *z* position of the animal into their respective phases (i.e. integral of the above two dynamic equations). The firing activity of azimuth PI neurons show band like activity in 3D space. An example of such a 3D azimuth band for an azimuth PI neuron of 303° is shown as Supplementary Figure 8 (XY projection of the same is shown right to the figure):

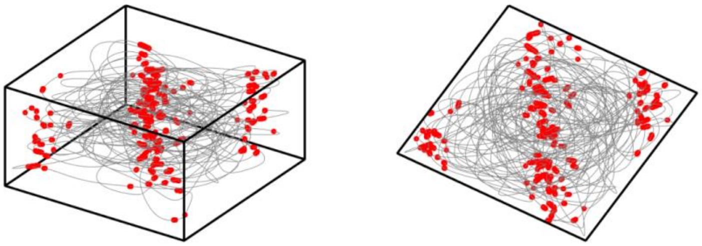

Supplementary Figure 8: 3D band like activity of the azimuth PI neuron (of 303°). The right panel shows the XY projection of the same.

The firing activity of pitch PI neurons also show band like activity in 3D space. An example of such a 3D pitch band for a pitch PI neuron of 90° is shown as Supplementary Figure 9 (XZ projection of the same is shown right to the figure):

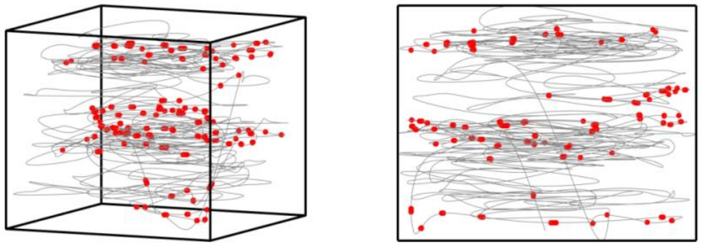

Supplementary Figure 9: 3D band like activity of the Pitch PI neuron (of 90°). The right panel shows the XZ projection of the same.

Anti-hebbian network neurons combine these bands in a certain fashion to give rise to a spectrum of 3D spatial representations. For example, a combination of three 3D bands that have an angular difference of 120° to each other gives rise to columnar hexagonal grids as shown as Supplementary Figure 10.

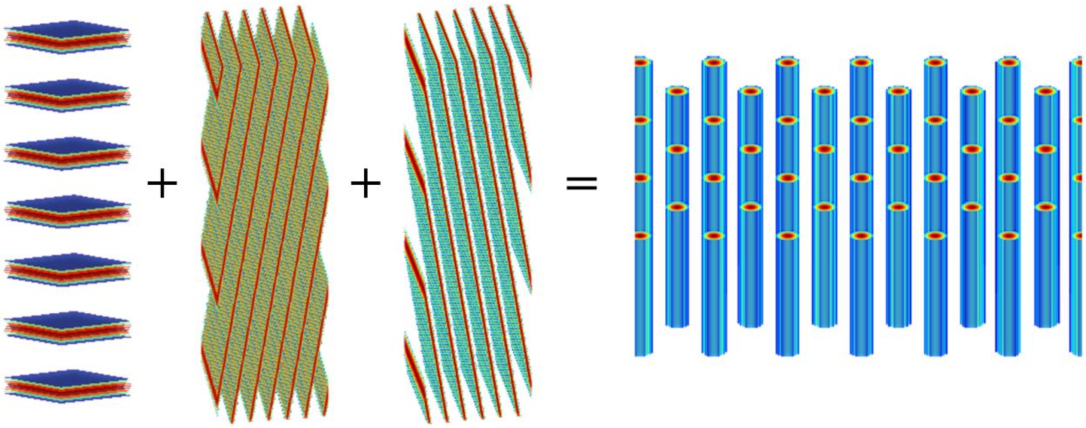

Supplementary Figure 10: Depiction of the linear combination of three 3D bands which are at 120° to each other. This combination give rise to columnar hexagonal representations.

If the combination is between the bands that have an angular difference of 109.5° to each other, it gives rise to FCC lattice structure grids as shown below (Supplementary Figure 11).

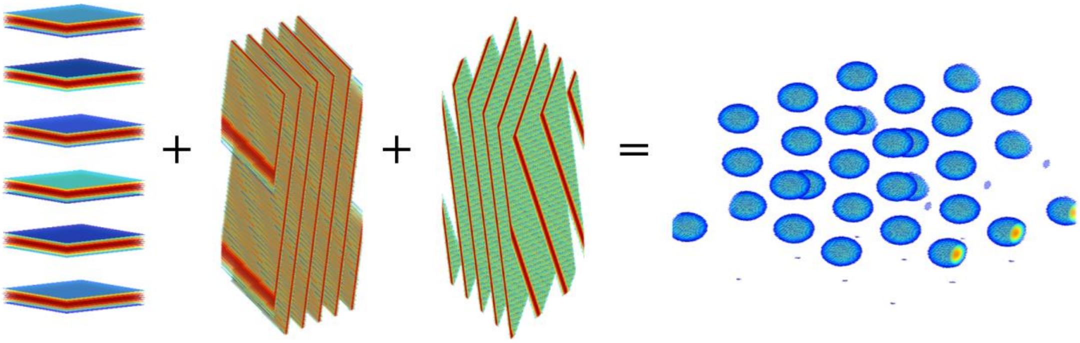

Supplementary Figure 11: Depiction of the linear combination of three 3D bands which are at 109.5° to each other. This combination further give rise to FCC lattice representations.

b. Dependence on initial position of the animal

The spatial representations from the model depend on the initial position of the animal on the trajectory. To make it clear let us consider the phase dynamics of an azimuth PI oscillatory neuron corresponding to 0° as given below.

$$\dot{\theta}_{\mathrm{Az}} = \omega + \beta\Delta x \qquad \text{------(4)}$$

Integrating the above equation from the starting time t = 0 up to the current time $T$ as shown below:

$$\int_{o}^{T} \dot{\theta}_{\mathrm{Az}}\, dt = \int_{0}^{T} (\omega + \beta\Delta x)\, dt$$

$$\left[\theta_{\mathrm{Az}}(t)\right]_0^T = \left[\omega t + \beta x(t)\right]_0^T$$

$$\theta_{\mathrm{Az}}(T)\text{-}\theta_{\mathrm{Az}}(0) = \omega T + \beta\left[x(T) - x(0)\right]$$

$$\theta_{\mathrm{Az}}(T) = \theta_{\mathrm{Az}}(0) + \omega t + \beta\left[x(T) - x(0)\right] \text{-------------(5)}$$

(Similar analysis as shown above holds true for the pitch path integration neurons too and we are not showing it here).

It is evident from the above Supplementary equation (5) that the phase of the oscillation at time $(T)$ depends on the initial phase and also the initial position of the animal. Without loss of generality if we assume that the initial phase of the oscillator is at zero, then the phase of the temporal oscillation solely depends on the initial position of the animal. Now assume two trials (i.e. two different trajectory of the animal) where the initial position of the animal is different for these two trials. A 3D band still retains its qualitative nature (i.e. 3D band will still be a 3D band), but there will be a spatial offset at which these bands occur for the two trajectories (as shown in Supplementary equation 5). Since the downstream spatial representations are the linear combinations of these 3D bands, then the same principle applies to them i.e. across the trials they remap but still retain its spatial firing pattern. This is a unique feature of the path integration system and visual inputs may possibly correct this phase offset and offer higher stability (less remapping) of spatial representations across the trials.

**Supplementary Note 5: Frequency variation analysis**

In the simulation of the model, we specifically choose a value of 0.5 Hz for the path integration oscillators based on the bat neuronal data obtained from [1,4,5]. However, the following analysis is based on treating the frequency as a free parameter of the model to check its influence on the spatial representations.

To analyze how the frequency parameter affects globally on the spatial cells we did two analyses as explained below:

1. We analyzed the influence of the frequency parameter by estimating the percentage of spatial cells formed in the model (by computing the spatial information index of each neuron) with respect to the frequency of the oscillations (varying from 0.5 Hz to 20 Hz with a resolution of 0.5 Hz). The hypothesis of this analysis is that, if the frequency parameter has a significant role in spatial encoding then a change in this parameter should influence the distribution of the formed spatial cells in the model. The plot below (Supplementary Figure 12) shows the result of the aforementioned analysis (obtained after training the network 10 times, each time re-initializing the weight connections, for each frequency. The average and standard deviation of the % of spatial cells formed for each frequency are used to generate the shaded plot shown below).

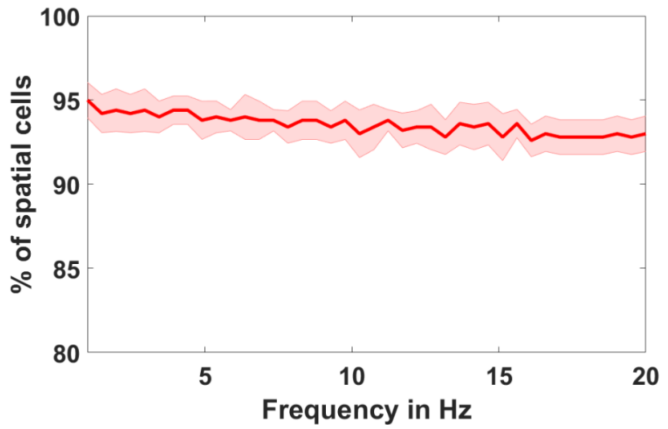


Supplementary Figure 12: Influence of oscillator frequency on the number of spatial cells formed. Solid red line shows the average % of spatial cells formed in the model and the light red shade shows the standard deviation. It is evident from the figure that the percentage of spatial cells formed in the model has only a slight decrease (2%) between the lower and upper limit of the frequency of the oscillator.

There occurs only a very slight decrease in the % of spatial cells (2%) between the lower limit frequency (0.5 Hz) and upper limit frequency (20 Hz). We envisage that the reason for this must be due to the neural learning mechanism in the anti-Hebbian network. To make it clearer, consider Supplementary equations (2) and (3) that show the encoding of position variable into the phase of the temporal oscillations. Since the afferent weight connections of the anti-Hebbian network are updated using the Hebbian rule, connections from those PI neurons that are active at the same time or in other words that are in-phase are enhanced. Also, all the oscillators in the model essentially use the same frequency ($\omega$ in Supplementary equations 2 and 3), hence it captures no spatial variance/information. The spatial variance comes from that part of the phase

dynamics which carry the positional information (Supplementary equations 2 and 3). Hence, in the phase dynamics, the term that carries the positional information is more critical than the absolute frequency of the oscillations ($\omega$ term in Supplementary equations 2 and 3). Hence, even if the frequency is changed, the network is endowed with the positional (spatial) information from the phase dynamics of the PI oscillators and hence this may be the reason for the spatial cell formation irrespective of a change in the frequency of the oscillators.

2. 3D navigating bats are empirically shown to have lesser hippocampal LFP frequency compared to the land dwelling rodents [1,4,5], but the result from the previous analysis (i.e. % of spatial cells vs frequency) is counter intuitive. Hence, next we analyzed the relation between the spatial information index of the simulated neuron [2] as a function of the frequency of the path integration oscillators (for the same range of the frequency used for the previous analysis i.e. from 0.5 Hz to 20 Hz with a resolution of 0.5Hz). For this, we considered the maximum value of the spatial information index of all the neurons in the anti-Hebbian network and computed the average of this after training the network 10 times (i.e. re-initializing the weight connections 10 times). The following plot (Supplementary Figure 13) shows the result of this analysis.

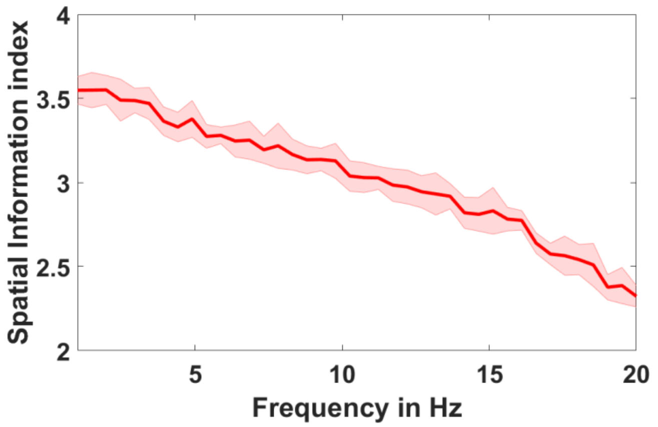


Supplementary Figure 13: Influence of oscillator frequency on the spatial information index of the neuron. Plot shows the spatial information index of the anti-Hebbian network neurons varying as a function of the frequency of the path integration oscillators. It is evident from the figure that there is a significant decrease in the spatial information index with an increase in the oscillatory frequency.

This result is interesting since this conveys that for higher frequency oscillations even if the model generates a significant number of spatial cells (which is evident from Supplementary Figure 12) the spatial information index is lesser for those cells. In other words, lower frequency oscillations provide spatial cells that convey more information about the space (higher spatial index). The possible reason for the aforementioned phenomena could be attributed to the fact that as the frequency of the oscillation decreases, the azimuth and pitch path integration oscillators come in-phase with a comparatively larger temporal scale compared to the case of higher frequency oscillations. This can possibly increase the spatial scale of the 3D bands (because the position is encoded into the phase of the temporal oscillations) and the downstream spatial cells could possibly have more localized spatial activity (owing to an increase in the scale of the 3D bands) and this more localized neural activity could possibly increase the corresponding spatial information index of the respective neuron. Since 3D navigation is more sophisticated than 2D navigation (because more spatial variables are involved during flight), this may

need spatial cells that convey more spatial information and it may be due to this reason that the oscillation frequency is at a lower regime for flying mammals like bats [1,4,5]. Another modeling study conducted by Hoffman et al (2016) supports the need for lower frequency oscillations in flying animals, where they show how suppression of theta rhythm enhances the place cell map formation in 3D space [6].

**Supplementary Note 6: Simulation result after training the animal on the vertical plane**

In the manuscript we showed the formation of stripe like representations for the grid cells on the vertical wall (Figure 4G). This resulted when the model was trained while the animal navigated on the horizontal plane and then tested on the vertical plane/wall. Now the question that arises naturally is that what happens if the animal is trained first on the vertical wall and then tested on the horizontal wall? To answer this, an additional simulation was done where we trained the network while the animal climbed the vertical wall with a restricted pitch distribution (the same distribution that gave rise to stripes Figure 5A) and tested while it moved freely on the horizontal floor (the same trajectory statistics that gave rise to hexagons in the previous case). The hypothesis is that if the representation does not depend on the trajectory statistics, after training, the network should essentially produce grids on the vertical wall (irrespective of the skewed pitch distribution).

However, the result shows that even if the network was trained as the animal moved on the vertical wall, the resulting representations were still stripes (Supplementary Figure 14; starting from the left side of the figure, the first two figures show the firing field and the autocorrelation map respectively). Testing the network on the horizontal floor gave rise to clear grids as shown below (Supplementary Figure 14; starting from the left side of the figure, third and fourth figures show the firing field and the autocorrelation map respectively). This reinforces the possibility that the stripe/hexagonal representation could be the result of movement pattern also and not just synaptic adaptation. This is in fact supported by Hayman et al (2011) empirical study such that, when the rat was rewarded only if it climbed the vertical wall (i.e. training the animal to navigate on the vertical wall), the representations were still stripes [7] which accords with the aforementioned result from this additional simulation.

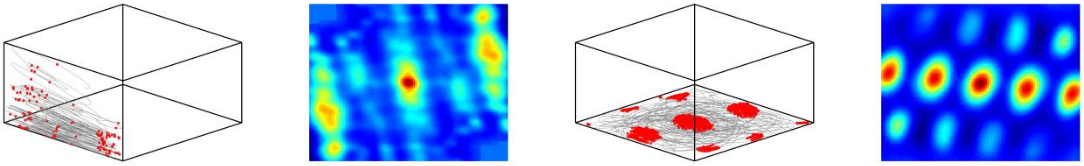

Supplementary Figure 14: Grid representations obtained after training the model while the animal climbed over the vertical wall. From left side of the figure: Firing field of the neuron on the vertical wall (First figure), Autocorrelation map of the representation on the vertical wall (Second figure), Firing field of the neuron on the horizontal plane (Third figure), Autocorrelation map of the representation on the horizontal plane (Fourth figure).

**Supplementary Note 7: Additional spatial representations from the model**

Here we include four additional spatial representations. Out of these four representations, two representations show inclined plane cell activity (Supplementary Figures 15A-B). The motivation of showing these two figures is to convey the fact that plane cells described in the manuscript need not always have horizontal firing fields. Supplementary Figures 15C-D show the rate maps of two cells that come under the category of Other Spatial Cell (OSC) band. Despite of the higher spatial information index they carry, they do not follow any particular spatial descriptor like grid score/border score/ plane score. As mentioned in the manuscript, this reminds the result from Diehl et al (2017), where neurons that carry relevant spatial information index but with atypical non-grid structure were reported from the medial entorhinal cortex of the rat [8] and these may be possibly the 3D counterparts of the same.

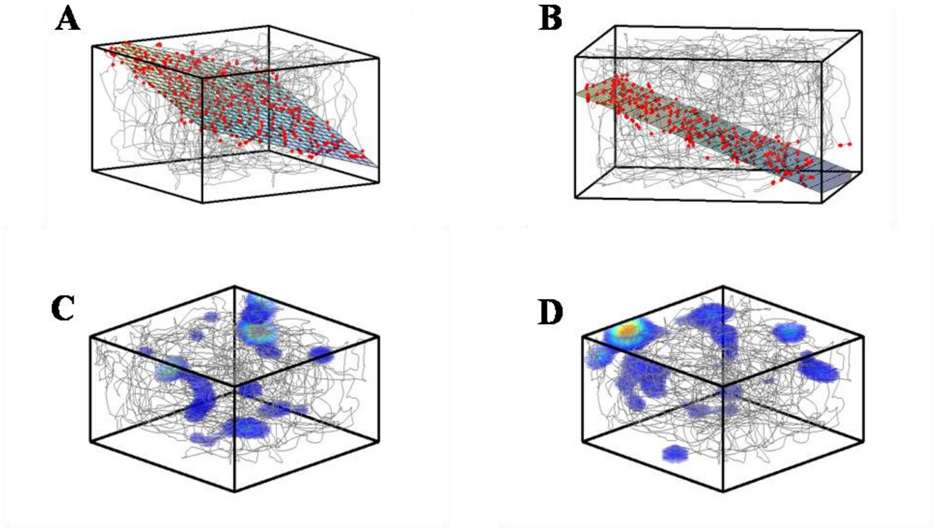


Supplementary Figure 15: Additional spatial representations from the model. (A) – (B) Inclined plane cells shown along with the plane fitted to their 3D firing fields. (C)-(D) rate maps of two Other Spatial Cell (OSC) with significant spatial information, but no typical spatial descriptor. Gray curves in the figures show the trajectory traversed by the animal.

**Supplementary References**


1 Yartsev, M. M. & Ulanovsky, N. Representation of three-dimensional space in the hippocampus of flying bats. *Science* **340**, 367-372, doi:10.1126/science.1235338 (2013).

2 Skaggs, W. E., McNaughton, B. L. & Gothard, K. M. in *Advances in neural information processing systems.* 1030-1037.

3 Stella, F. & Treves, A. The self-organization of grid cells in 3D. *Elife* **4**, e05913, doi:10.7554/eLife.05913 (2015).

4 Heys, J. G., MacLeod, K. M., Moss, C. F. & Hasselmo, M. E. Bat and rat neurons differ in theta-frequency resonance despite similar coding of space. *Science* **340**, 363-367, doi:10.1126/science.1233831 (2013).

5 Yartsev, M. M., Witter, M. P. & Ulanovsky, N. Grid cells without theta oscillations in the entorhinal cortex of bats. *Nature* **479**, 103-107, doi:10.1038/nature10583 (2011).

6 Hoffman, K., Babichev, A. & Dabaghian, Y. Topological mapping of space in bat hippocampus. *arXiv preprint arXiv:1601.04253* (2016).

7 Hayman, R., Verriotis, M. A., Jovalekic, A., Fenton, A. A. & Jeffery, K. J. Anisotropic encoding of three-dimensional space by place cells and grid cells. *Nat Neurosci* **14**, 1182-1188, doi:10.1038/nn.2892 (2011).

8 Diehl, G. W., Hon, O. J., Leutgeb, S. & Leutgeb, J. K. Grid and Nongrid Cells in Medial Entorhinal Cortex Represent Spatial Location and Environmental Features with Complementary Coding Schemes. *Neuron* **94**, 83-92 e86, doi:10.1016/j.neuron.2017.03.004 (2017).
